# Comprehensive Analysis of the AP2/ERF Superfamily Identifies Key Genes Related to Various Stress Responses in Olive Tree (*Olea europaea* L.)

**DOI:** 10.3390/cimb48020183

**Published:** 2026-02-05

**Authors:** Erli Niu, Song Gao, Mengyun Ren, Wei Wang, Qian Zhao, Ying Fu

**Affiliations:** 1 Institute of Crops and Nuclear Technology Utilization, Zhejiang Academy of Agricultural Sciences, Hangzhou 310021, China; niuerli@zaas.ac.cn (E.N.); gaos@zaas.ac.cn (S.G.); renmengyun@zaas.ac.cn (M.R.); 2 School of Biological and Chemical Engineering, Zhejiang University of Science and Technology, Hangzhou 310023, China; wangwei5228345@zust.edu.cn

**Keywords:** olive (*Olea europaea* L.), AP2/ERF, genome-wide identification, expression profiling analysis, stress response

## Abstract

The AP2/ERF superfamily is a key class of transcription factors involved in plant responses to various stresses. As an ancient species, the olive tree (*Olea europaea* L.) exhibits considerable stress tolerance and wide adaptability. In this study, we identified 348 AP2/ERF genes in the cultivated olive variety ‘Arbequina’ at the whole-genome level. According to protein sequence alignments and phylogenetic analyses via the Maximum Likelihood method, these genes were classified into four major families: AP2, ERF/DREB, RAV, and Soloist. The ERF/DREB family was further divided into DREB and ERF subfamilies, each encompassing six groups (A1–A6 and B1–B6), with the ERF subfamily being the largest. Members of each group exhibited relatively consistent gene structures and domain/motif compositions of their encoded proteins; however, the distribution of *cis*-elements and expression patterns varied. Each AP2/ERF gene contained 12 light-responsive, three MeJA-responsive, three ABA-responsive, two anaerobic induction, and one MYB binding site on average. With the threshold of *p* value < 0.5, control TPM > 0, and |log_2_(fold change)| > 0, 50 candidate genes were simultaneously up-regulated (30) or down-regulated (20) under four stress treatments (acid–aluminum, cold, disease, and wound), among which nine showed potential protein–protein interactions. This study provides a comprehensive genomic characterization of the AP2/ERF family in olive and identifies key candidate stress-responsive genes, establishing a foundation for future functional studies on the molecular mechanisms of stress adaptation in the olive tree.

## 1. Introduction

Olive tree (*Olea europaea* L.), a long-lived and economically important evergreen tree belonging to the Oleaceae family, plays a key role in both agricultural and ecological systems [1]. Olive oil, extracted from olive fruits, is a cornerstone of the Mediterranean diet and rich in monounsaturated fatty acids, particularly oleic acid, which contributes to cardiovascular health and oxidative stability [2]. Native to the Mediterranean region and cultivated for over 6000 years, this species has evolved a suite of physiological and morphological adaptations that allow it to thrive under challenging environmental conditions characterized by aridity, poor soil fertility, and substantial seasonal temperature variations [1,3,4]. Beyond their observable morphological traits, such as well-developed root systems and waxy leaves, unraveling the molecular genetic basis of this resilience is crucial for enhancing sustainable olive cultivation. In this case, transcription factors (TFs) act as the main regulatory factors that can help plants cope with biotic and abiotic stresses, such as pathogen attacks, drought, and extreme temperatures or soils [5,6,7,8].

Among plant-specific TFs, the APETALA2/ethylene response factor (AP2/ERF) superfamily is one of the most prominent and versatile families, and has been identified ubiquitously in *Arabidopsis thaliana*, rice (*Oryza sativa*), maize (*Zea mays*), soybean (*Glycine max*), and Siberian apricot (*Prunus sibirica*) [9,10,11,12]. Its members are defined by the presence of at least one highly conserved AP2 DNA-binding domain. This superfamily is categorized into four major families based on domain architecture: the AP2 family (containing one or two AP2 domains), the Related to ABI3/VP1 (RAV) family (possessing an AP2 domain and a B3 domain), the ERF/dehydration-responsive element binding proteins (ERF/DREB) family (featuring a single AP2 domain), and the Soloist family (distinct members with a AP2 domain and low sequence similarity to others) [13,14,15]. The ERF/DREB family is further subdivided into the DREB (A1–A6 groups) and ERF (B1–B6 groups) subfamilies, which primarily bind to dehydration response element (DRE)/C-repeat (CRT) and Ethylene response element (ERE)/GCC-box cis-elements, respectively. In addition, the AP2/ERF members may contain various *cis*-elements, including TTG, hypoxia-responsive promoter element (HRPE), and others [15].

The AP2/ERF superfamily plays diverse roles across plant growth and morphological development, responses to external environmental stimuli, and hormone signaling. For instance, AP2-like genes, targeted by miR172, critically determine floral organ integrity and control flowering time in orchid *Cymbidium ensifolium* and ornamental gloxinia [16,17]. In cotton, *GhERF108* interacts with auxin response factors *GhARF7-1* and *GhARF7-2*, thereby activating a downstream MYB gene *GhMYBL1*, and ultimately promoting the secondary cell wall biosynthesis [18]. The AP2/ERF superfamily is particularly renowned for its central role in mediating plant adaptation to a wide spectrum of environmental stresses, particularly the DREB/ERF family. For example, the base-edited *OsERF52* variants confer improved chilling tolerance with no significant yield loss in rice [19]. Furthermore, members like AtERF71 in *Arabidopsis* integrate responses to multiple stresses, including osmotic stress and hypoxia, with overexpression leading to improved tolerance to salt, mannitol, flooding, and methyl viologen (MV) [20]. The regulatory prowess of AP2/ERF TFs is amplified through their extensive interplay with multiple plant hormone signaling pathways. They act as critical nodes in networks involving ABA, ethylene, gibberellin (GA), brassinosteroid (BR), and cytokinin, jasmonic acid (JA), auxin (indole-3-acetic acid, IAA) [13,14,15,21,22]. This crosstalk allows AP2/ERF factors to integrate diverse endogenous and exogenous signals, fine-tuning the balance between growth and defense. For instance, in Populus, *PtoERF15* and its target *PtMYC2b* enhance drought tolerance via the JA signal pathway, and also cause the anatomical changes in vessel size, density, and cell wall thickness [21]. The AP2/ERF superfamily genes are abundant and functionally diverse in different species, and they have always been a research focus, with ongoing studies uncovering more regulatory relationships.

Given their established importance in development and stress adaptation, AP2/ERF genes have been extensively characterized among various plants. However, a comprehensive analysis of this superfamily, including the genome-wide identification and transcriptomic responses across multiple stresses, is still lacking for these long-lived, stress-tolerant perennial trees. Previous studies on olive have primarily focused on variety selection, cultivation, and oil quality, with systematic characterization of its TF repertoire lagging behind. Identifying the full complement of AP2/ERF genes in the olive genome, analyzing their phylogenetic relationships, gene structures, conserved motifs, promoter *cis*-elements, and response to adversity, constitutes a critical foundational step. Such a study is necessary to unlock the potential of these key regulatory genes. It will provide a valuable resource for understanding the molecular basis of olive’s unique adaptability and identify candidate genes for future functional validation and breeding strategies aimed at enhancing stress tolerance and productivity in olive and potentially other woody perennials.

## 2. Materials and Methods

### 2.1. Identification and Characterization of AP2/ERF Genes in Olive

Genome data of the widely adaptable olive variety ‘Arbequina’ were retrieved from the genome warehouse in the national genomics data center (No. PRJCA003222, https://ngdc.cncb.ac.cn/gwh/Assembly/10300/show, last accessed on 25 March 2024) [23]. The AP2 domain profile (PF00847) of the AP2/ERF superfamily was downloaded from the Pfam database (http://pfam.xfam.org/, last accessed on 25 March 2024) [24]. To identify AP2/ERF genes in olives, HMMER 3.3.2 [25] with an E-value threshold of 1 × 10^−5^ was employed to search the olive proteome database using PF00847 as the query, with reference to *A. thaliana* AP2/ERF sequences [8]. All the retrieved sequences (requiring a length of no less than 60 amino acids) and the sequences exceeding the threshold but containing complete domain were further validated for conserved AP2 domains using SMART v9 (http://smart.embl-heidelberg.de/, last accessed on 13 August 2024) [26] and the NCBI conserved domains database (CDD, https://www.ncbi.nlm.nih.gov/Structure/cdd/wrpsb.cgi, last accessed on 13 August 2024) [27]. For the sequences with incomplete (*GWHGAOPM039967*) or duplicated structural domains (*GWHGAOPM002452* and *GWHGAOPM002474*), other olive genome ‘Farga’ (https://denovo.cnag.cat/olive, last accessed on 15 April 2024) [28] was used for correction. For multiple splice variants, only the longest transcript was retained as the representative sequence for subsequent analysis to avoid redundancy.

### 2.2. Characterization and Subcellular Localization of the Proteins Encoded by AP2/ERF Genes

Biochemical characteristics, including molecular weight (MW) and isoelectric point (pI), of AP2/ERF-encoded proteins were analyzed using ExPASy ProtParam server (https://web.expasy.org/protparam/, last accessed on 13 August 2024) [29]. Subcellular locations of these proteins were predicted in parallel with two independent tools: WoLF PSORT program (score of ≥4, https://psort.hgc.jp/, last accessed on 14 August 2024) and Softberry ProtComp 9.0 (score of >6.0, http://www.softberry.com/berry.phtml, last accessed on 14 August 2024). A localization was assigned only when the results from both tools were in agreement, and the conflicting localization was further adjudicated using Cell-PLoc 2.0 (http://www.csbio.sjtu.edu.cn/bioinf/Cell-PLoc-2/, last accessed on 14 August 2024) and the prediction of orthologous genes in *A. thaliana*.

### 2.3. Phylogenetic Analysis and Classification

Phylogenetic analysis of AP2/ERF proteins from olive and *A. thaliana* was performed as follows: Sequences of AP2/ERF family proteins in *A. thaliana* were retrieved from the *Arabidopsis* Information Resource (TAIR, http://www.arabidopsis.org, last accessed on 25 March 2024) [30]. Multiple sequence alignment of the 495 amino acid sequences was conducted using Clustal X 2.1 [31]. The alignment was trimmed to eliminate all positions with less than 95% site coverage, resulting in a final dataset of 49 amino acid positions for analysis. Phylogenetic tree reconstruction was performed via the Maximum Likelihood method with the Jones–Taylor–Thornton matrix-based model in MEGA 7.0 software [32]. The tree with the highest log likelihood (−9448.27) is presented and was rooted using the *A. thaliana* Soloist (AT4G13040) protein as the outgroup, following established classification criteria [9,33]. Branch support was assessed with 1000 bootstrap replicates.

### 2.4. Chromosome Distribution and Gene Duplication Analysis

Chromosomal localization data of AP2/ERF genes were retrieved from olive genome annotations and visualized via the GTF/GFF parser module in TBtools-II [34], using its gene location visualization function. Tandem duplication was defined as genes separated by ten or fewer genes on the same chromosome. The segmental duplication was identified using the One Step MCScanX algorithm and visualized using the Advanced Circos module in TBtools-II [34].

### 2.5. Conserved Domain and Structural Analyses

Conserved motifs of AP2/ERF proteins were predicted using MEME Suite 5.5.9 (https://meme-suite.org/meme/index.html, last accessed on 13 August 2024) [35]. The analysis was configured to identify up to 10 distinct motifs, based on preliminary runs and aligned with the AP2/ERF proteins in *A. thaliana* [8,33]. The search was performed with an E-value threshold of <1 × 10^−5^, requiring each identified motif to be statistically significant. More than 80% of genes within each group contain motifs that are considered characteristic motifs of that group. Conserved domains in these proteins were identified via NCBI CDD search [27] and SMART tool [26] with default parameters. Exon/intron structures of AP2/ERF genes were visualized based on olive genome annotation data [23].

### 2.6. Prediction of Cis-Elements in the Promoter Regions

The 2000 bp upstream genomic sequence of the 5′ end of the open reading frame was extracted as the putative promoter region and used to predict the *cis*-elements via the GTF/GFF3 Sequences Extract program in TBtools-II [34]. *Cis*-elements in the promoter regions of AP2/ERF genes were predicted using the PlantCARE database (https://bioinformatics.psb.ugent.be/webtools/plantcare/html/, last accessed on 4 September 2024) [36]. The hormone- and environmental factors-related *cis*-elements were counted and visualized using the Simple BioSequence Viewer program in TBtools-II [34].

### 2.7. Response Patterns of AP2/ERF Genes to Different Stresses in Olive

The publicly transcriptome databases, including the profiles PRJNA1299032 (acid–aluminum stress) and PRJNA256033 (cold, disease-*Verticillium dahlia* and wound stress), were downloaded from NCBI (https://www.ncbi.nlm.nih.gov/, last accessed on 9 September 2024). These data were reanalyzed and generated an average of 55.64 million raw reads per sample. Raw reads were trimmed to remove adapters, low quality bases (Q < 20), and short reads (<50 bp) using fastp v0.22.0 [37]. The clean reads were then aligned to the ‘Arbequina’ olive genome [23] using HISAT v2.2.0 [38], with an average alignment rate of 87.1% after excluding four outlier data points. To identify differentially expressed genes (DEGs), the raw read counts for each gene were used as input for DESeq2, with the design matrix incorporating key experimental factors including stress type, tissue, and treatment time. Genes with an adjusted *p* value (FDR) < 0.05 were considered statistically significant. For visualization of expression levels, transcript abundance was also quantified as transcripts per million (TPM) using Cufflinks 2.2.1 (http://cufflinks.cbcb.umd.edu/, last accessed on 9 September 2024). The final set of DEGs for biological interpretation was further refined by requiring a |log_2_(fold change)| > 0. The representative time points were selected for comparative analysis and heatmap generation based on preliminary examination of the transcriptional changes under different stress treatments. Specifically, for acid–aluminum and disease treatments, root samples were selected at 48 h post-treatment, while for cold stress (leaves) and wound stress (roots), samples were selected 24 h post-treatment. Pearson’s correlation coefficient and PCA analysis of different profiles are shown in Figure S1.

### 2.8. Protein Interaction Networks and Gene Ontology (GO) Enrichment Analysis

Protein–protein interaction (PPI) and GO enrichment analysis were conducted in the STRING database 12.0 (https://cn.string-db.org/, last accessed on 10 June 2025) [39]. Due to the lack of olive data, we referred to the *A. thaliana* dataset and searched for the interaction partners of 50 key AP2/ERF genes. Specifically, the protein sequences of candidate genes were queried against the *A. thaliana* proteome using BLASTp program with a stringent E-value cutoff of 1 × 10^−40^, and *A. thaliana* protein hits with sequence similarity > 45% were selected as query entries for subsequent PPI network prediction with the minimum required interaction score was set to high confidence (0.700) and FDR was set to high stringency (1%). Functional enrichment analysis for the resulting interaction network was performed using default settings in STRING 12.0 [39].

## 3. Results

### 3.1. Genome-Wide Identification and Characterization of AP2/ERF Superfamily in Olive Tree

Using the AP2 domain profile (PF00847) of the AP2/ERF gene family from the Pfam database [24], we performed a Hidden Markov Model (HMM) search against the protein databases of ‘Arbequina’, a widely adaptable, high-yielding olive cultivar [23]. After validating domains with the online tools SMART v9 [26] and NCBI CDD [27], a total of 348 AP2/ERF genes were obtained in olive (Table 1 and Table S1).

The polypeptides encoded by AP2/ERF genes had a mean length of 270 amino acids and a mean MW of 30.1 kDa (Table 1). The shortest sequence, *GWHGAOPM010521* (79 amino acids), encoded the protein with the smallest MW (9.1 kDa), while the longest sequence, *GWHGAOPM041320* (650 amino acids), encoded the protein with the largest MW (72.2 kDa) (Table 1). Charge distribution analysis showed pI variations with an average of 6.98 (4.19–11.03) across the polypeptides: *GWHGAOPM017031* represented the most acidic isoform (pI 4.19), and *GWHGAOPM040516* represented the most basic isoform (pI 11.03) (Table 1). Furthermore, 32 AP2/ERF genes had instability indices < 40 (encoding stable proteins), while the remaining 316 had instability indices > 40 (encoding unstable proteins) (Table 1). Subcellular location prediction revealed that 83.91% (*n* = 292), 12.93% (*n* = 45), and 2.87% (*n* = 10) of AP2/ERF proteins localized exclusively to the nucleus, to both the nucleus and cytoplasm, and exclusively to the cytoplasm, respectively. One encoded protein (*GWHGAOPM011467*) exhibited localization to both the cytoplasm and the chloroplast.

### 3.2. Phylogenetic Analysis and Classification of AP2/ERF Genes

**Figure 1:**
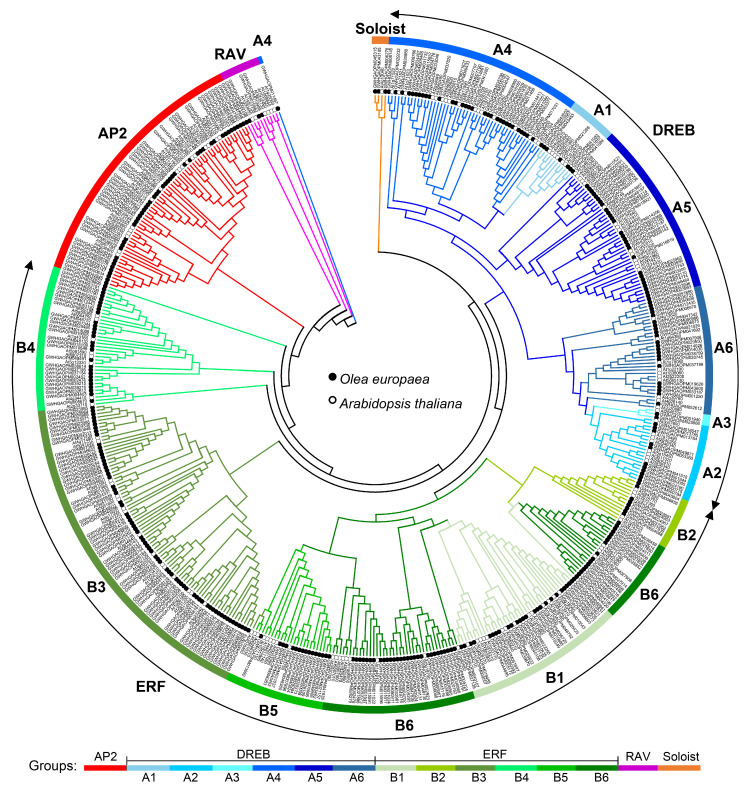
Phylogenetic tree of the AP2/ERF superfamily in olive and *Arabidopsis thaliana*. Full-length amino acid sequences of the AP2/ERF superfamily were obtained to conduct the phylogenetic tree via the Maximum Likelihood method based on the Jones–Taylor–Thornton (JTT) matrix model using MEGA 7.0 software. Solid and open circles represent genes from olive and *A. thaliana*, respectively. Different groups were marked with lines of different colors.

To elucidate the phylogenetic relationships and classification of AP2/ERF genes in olive, a comparative genomic analysis was conducted using all AP2/ERF protein sequences from olive and *A. thaliana* (Table 1, Figure 1, Supplementary Material File S1, Table S1). Phylogenetic reconstruction using the maximum likelihood method allowed for the systematic classification of olive AP2/ERF members, following the established *A. thaliana* classification criteria [9,33].

The phylogenetic tree was resolved into four conserved families (AP2, ERF/DREB, RAV, and Soloist) within the olive AP2/ERF superfamily and revealed a disproportionate distribution among subfamilies (Figure 1, Table 1). The AP2 family consisted of 46 members (13.2%). The ERF/DREB family emerged as the largest group (*n* = 295, 84.8%) and was further divided into two subfamilies: DREB (*n* = 103, 29.6%) and ERF (*n* = 192, 55.2%) (Table 1, Figure 1). The DREB subfamily was further classified into six functional groups (A1–A6), containing 6, 11, 2, 33, 29, and 22 members, respectively. Similarly, the ERF subfamily also contained six groups (B1–B6), with 26, 8, 68, 28, 16, and 46 genes, respectively. The RAV and Soloist families comprised 4 and 3 genes, respectively (Table 1, Figure 1). This hierarchical classification system offers critical insights into the evolutionary conservation and potential functional specialization of the olive AP2/ERF superfamily.

### 3.3. Chromosome Distribution and Gene Duplication of AP2/ERF Genes

**Figure 2:**
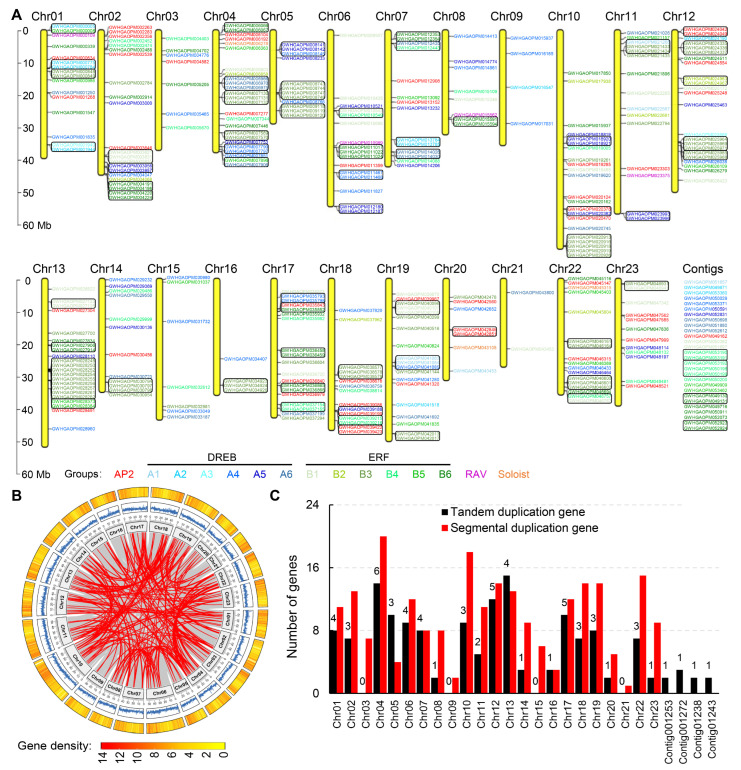
Chromosome distribution and gene duplication of 348 AP2/ERF genes in olives. (**A**) Chromosome distribution and tandem duplication of AP2/ERF genes. Numbers of the 23 chromosomes are indicated at the top of each vertical yellow bar, and black boxes represent the tandem duplication gene pairs. Different colors of gene names represent the corresponding groups of the AP2/ERF superfamily. (**B**) Segmental duplication of AP2/ERF genes. Red lines represent the segmental duplication gene pairs, while gray lines depict all syntenic blocks in the olive genome. The inner circle shows the length of chromosomes, and blue lines indicate the GC content. Gene density is represented by a heat map (outer circle) and marked with red/yellow blocks. (**C**) The number of duplicate genes located on different chromosomes. The *X*-axis indicates the different chromosomes and contigs, and the *Y*-axis indicates the corresponding gene number of tandem duplication (black)and segmental duplication (red). The number of tandem duplication gene pairs is also shown in the black column.

Among the olive AP2/ERF genes, 28 were located to chromosomal scaffolds, while the remaining 320 were distributed unevenly across the 23 chromosomes with an average density of 3.09 Mb per gene (1.47–8.71 Mb) (Figure 2A). Five of these chromosomes harbored more than 20 genes each: chromosomes 4, 12, 2, 10, and 13, possessing 25, 23, 22, 22, and 22 genes, respectively (Figure 2A). In contrast, chromosomes 9 and 16 each contained 4 genes, while chromosome 21 harbored the fewest genes (*n* = 2).

An analysis of gene duplication within the AP2/ERF superfamily revealed the presence of 61 tandem duplication pairs (150 genes) and 201 segmental duplication pairs (229 genes) (Figure 2, Table S2). Among tandem gene pairs, there were 41 pairs of repeats between 2 genes, 17 pairs of repeats involving 3 genes (Figure 2A, Table S2). In addition, 1 tandem pair involved 4, 5, and 7 genes, which were located on Chr05, Chr10, and Chr13, respectively (Figure 2A, Table S2). Among segmental gene pairs, there were 119 genes showing no segmental collinear relationships with other AP2/ERF members, and 2, 3, 45, 66, and 113 genes were involved in 5, 4, 3, 2, and 1 segmental duplicated gene pairs, respectively (Figure 2B, Table S2). Regarding these gene duplication relationships, Chr13 and Chr04 participated in a large number of tandem repeat genes, with 15 and 14, respectively, while Chr04 and Chr10 had a large number of segmental repeat genes, with 20 and 18, respectively (Figure 2A–C, Table S2).

### 3.4. Conserved Domain of Encoded Proteins and Gene Structure Analyses of AP2/ERF Genes

**Figure 3:**
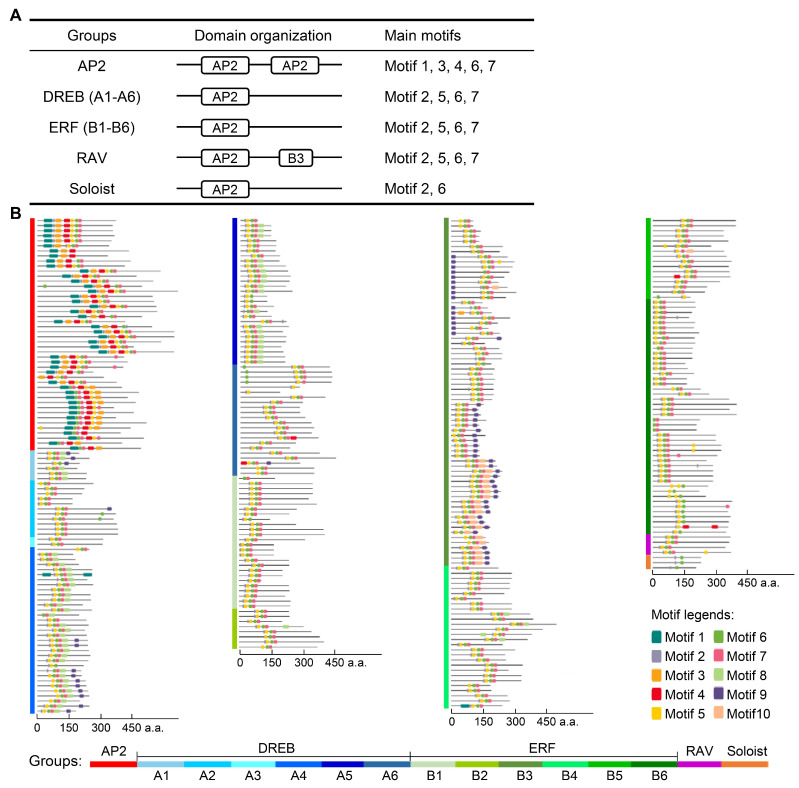
Conserved domains and motifs of AP2/ERF-encoded proteins in olive. (**A**) Domain organization and main motifs in different groups. Distinct conserved domains are represented by boxes. More than 80% of genes within each group contain motifs that are considered characteristic motifs of that group. The detailed domain and motif compositions of each gene are presented in Table S1. The diagrams of the conserved domains of the encoded proteins of each gene are presented in Figure S2. (**B**) Top ten motifs with the highest sequence similarity of AP2/ERF proteins. The motifs are represented by boxes of different colors. Different groups of AP2/ERF are presented in different color blocks on the left, and the gene names of corresponding proteins are listed in Table S1.

Analysis of conserved domains and motifs in AP2/ERF-encoded proteins revealed high intragroup similarity. AP2 subfamily proteins all contained two copies of the AP2 domain (Figure 3A and Figure S2). More than 80% of them had conserved motifs 1, 3, 4, 6, and 7, and 78.3% of genes have motif 5 (Figure 3A,B, Table S1). All proteins in DREB and ERF subfamilies contained one AP2 domain each (Figure 3A and Figure S2), and predominantly contained motifs 2, 5, 6, and 7 (>80% proteins), with 52.4% of DREB proteins containing additional selective motif 8 (Figure 3A,B, Table S1). RAV subfamily proteins also encompassed motifs 2, 5, 6, and 7, but each contained one AP2 domain at the N-terminus and a B3 functional domain (PF02362) at the C-terminus. Soloist subfamily proteins contained motifs 2 and 6, with one AP2 functional domain each (Figure 3 and Figure S2, Table S1).

**Figure 4:**
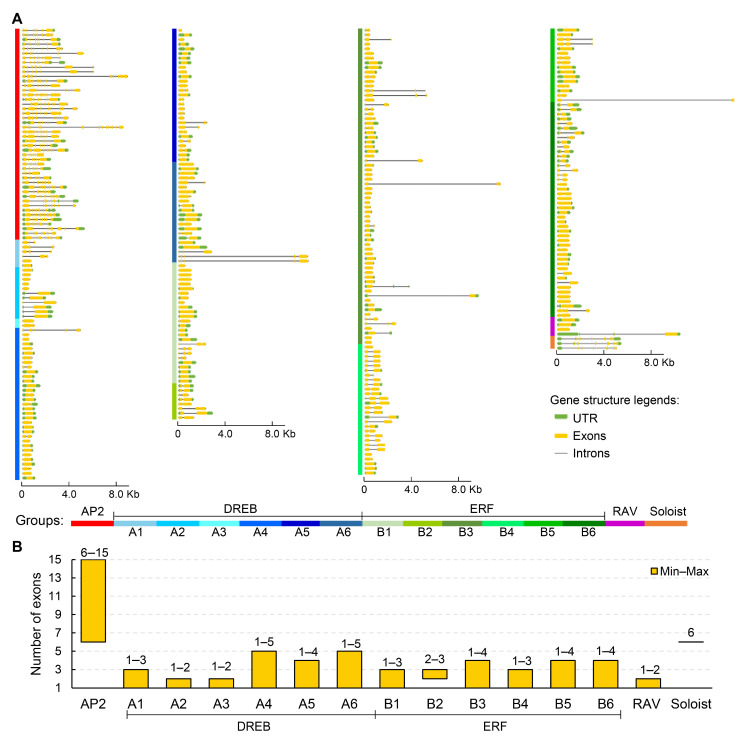
Gene structures of AP2/ERF members in the olive tree. (**A**) The diagram of gene structures. Untranslated regions (UTR), exons, and introns are indicated by the green box, yellow box, and gray lines, respectively. Different groups of AP2/ERF are presented in different color blocks on the left, and the gene names of corresponding proteins are listed in Table S1. (**B**) Statistics on the number of exons of different groups. The minimum–maximum (Min–Max) number of exons in different groups was statistically analyzed.

Moreover, AP2/ERF genes displayed distinct intragroup structural similarities (Figure 4A,B). For instance, AP2 subfamily genes were characterized by a relatively high number of exons/introns (6–15), with *GWHGAOPM039086* having the most exons. DREB and ERF subfamily genes had 1–5 and 1–4 exons, respectively. RAV subfamily genes had relatively simple structures: contained 1–2 exons, while all Soloist subfamily genes contained 6 exons (Figure 4A,B). In addition, according to genome annotation information, 163 genes were predicted to have UTRs among 348 AP2/ERF genes (Figure 4A).

### 3.5. Cis-Elements in the Promoters of AP2/ERF Genes

**Figure 5:**
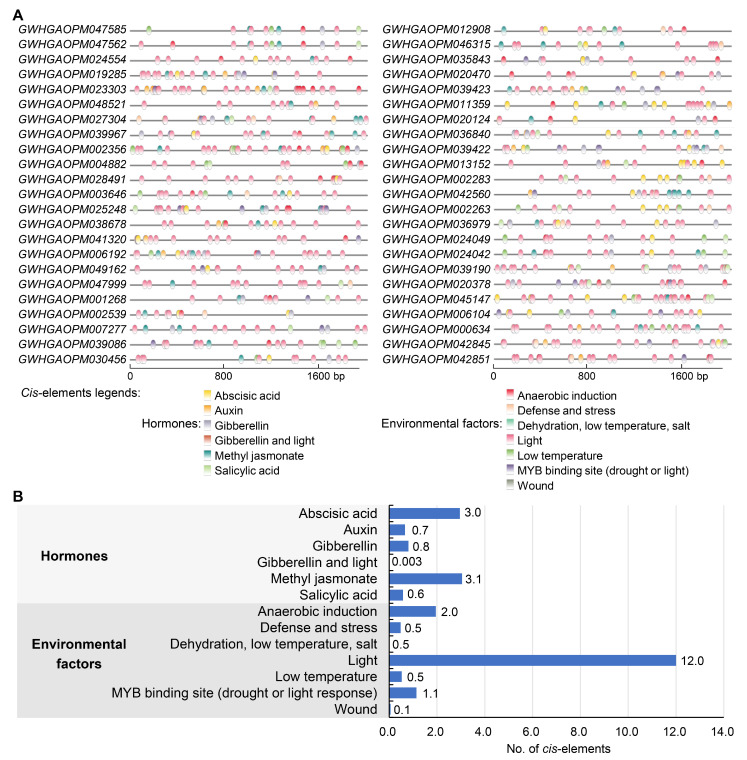
*Cis*-elements predicted in the promoters of AP2/ERF genes. (**A**) Distribution of different *cis*-elements: a case study of the AP2 subfamily. The 2000 bp sequence upstream of the 5′ end of the open reading frame of each gene was analyzed to predict the *cis*-elements. *Cis*-elements are represented by circles of different colors. The distribution of *cis*-elements in promoters of other subfamily members is shown in Figure S3. (**B**) Average statistics of different *cis*-elements per AP2/ERF gene. The graph and statistical values contain *cis*-elements related to hormones (abscisic acid, auxin, gibberellin, methyl jasmonate, and salicylic acid), and *cis*-elements related to environmental factors (anaerobic induction, defense and stress responses, dehydration, low temperature, salt, light, drought, and wound).

To further understand the potential functions of AP2/ERF genes in olive, the promoter sequences (2000 bp upstream) of 348 genes were analyzed (Figure 5A,B and Figure S3). Among these *cis*-elements, five were hormone-related: ABA, IAA, GA, methyl jasmonate (MeJA), and salicylic acid (SA). Additionally, some elements were associated with environmental factors, including anaerobic induction, defense and stress responses, dehydration, low temperature, salt, light, drought, and wound (Figure 5A and Figure S3). On average, each gene had 12 light-responsive, three MeJA-responsive, three ABA-responsive, two anaerobic induction, and one MYB binding site (drought or light response), respectively (Figure 5B and Figure S3). The light-responsive *cis*-elements mainly included G-box, box 4, GT1-motif, TCT-motif, and GATA-motif. MeJA-responsive *cis*-elements included CGTCA-motif and TGACG-motif, and the ABA-responsive and anaerobic induction -responsive *cis*-elements were ABRE and ARE, respectively. Moreover, some genes contained *cis*-elements like IAA, GA, SA, defense and stress responses, and low temperature (more than 0.5 per gene).

### 3.6. Response Patterns of AP2/ERF Genes to Different Stresses in the Olive Tree

**Figure 6:**
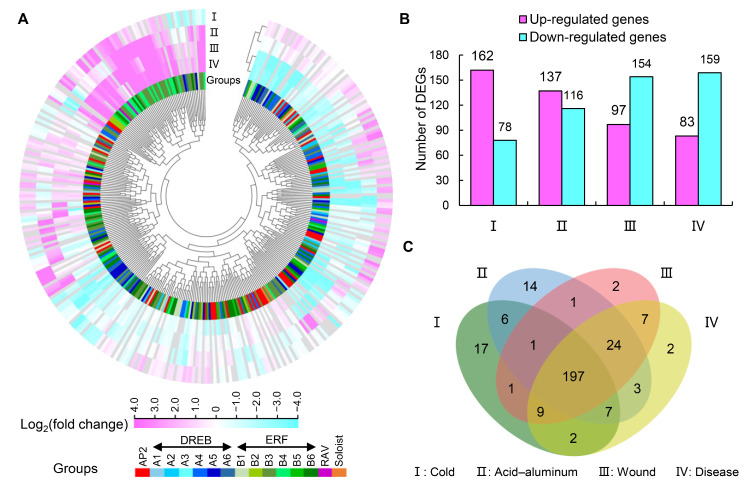
Expression changes in AP2/ERF genes under different stress treatments. (**A**) Heatmap of AP2/ERF genes. (**B**) Statistics of differentially expressed genes (DEGs). (**C**) Venn diagram of differentially expressed genes (DEGs). The stresses included cold (I), acid–aluminum (II), wound (III), and disease (IV). Expression changes are quantified as the log_2_ (fold change) of under stress vs. control. Gene groups are marked with different color blocks.

Using the available transcriptome data from the NCBI sequence read archive, we analyzed and explored the expression patterns of AP2/ERF genes under acid–aluminum, cold, disease, and wound stresses (Figure 6). Overall, AP2/ERF genes in olive showed similar response patterns only among genes with high sequence similarity, but did not have specific response patterns within each group (Figure 6). We hypothesized this was because, in addition to the conserved domains, AP2/ERF superfamily genes have functionally diverse motifs and *cis*-elements. Notably, 253 DEGs were identified after acid–aluminum treatment, with 137 up-regulated and 116 down-regulated (Figure 6A,B). *GWHGAOPM041144* (B3 group) and *GWHGAOPM003956* (A5 group) were the most strongly up-regulated and down-regulated in response to acid–aluminum stress, respectively. While 240 DEGs were found changed after cold stress, with 162 up-regulated and 78 down-regulated (Figure 6A,B). *GWHGAOPM002474* (B4 group) and *GWHGAOPM046694* (B6 group) were the most strongly up-regulated and down-regulated in response to this stress, respectively. After acid–aluminum and cold stress treatments, there were more up-regulated genes than down-regulated genes (Figure 6B). However, under wound stress (97 up-regulated and 154 down-regulated genes) and disease stress (83 up-regulated and 159 down-regulated genes), the number of down-regulated genes was higher than that of up-regulated genes (Figure 6B). Under wound and disease stresses, *GWHGAOPM034924* (B3 group) was most strongly up-regulated; *GWHGAOPM046315* (AP2 group) and *GWHGAOPM021268* (A1 group) were most strongly down-regulated, respectively. Overall, 197 DEGs were up-regulated or down-regulated under the four stress conditions (Figure 6C). Among these, 30 were universally up-regulated and 20 were universally down-regulated; 50 genes in total showed consistent directional change across all stresses. Additionally, 44, 56, and 115 genes were up-regulated by three, two, and one of these stresses, respectively, and 77, 72, and 52 genes were down-regulated by three, two, and one of these stresses, respectively.

### 3.7. Features of Key AP2/ERF Genes Responding to Different Stresses

**Figure 7:**
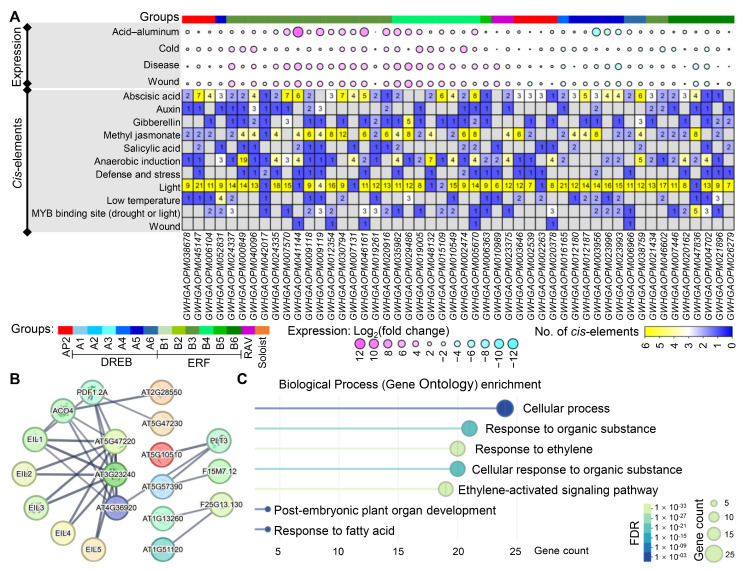
Comprehensive analysis of AP2/ERF genes affected by all four stresses: acid–aluminum, cold, disease, and wound. (**A**) Expression patterns and *cis*-elements of 50 candidate genes. The expression changes are displayed as log_2_(fold change). The counts of *cis*-elements related to hormones and environmental factors are shown, including abscisic acid, auxin, gibberellin, methyl jasmonate, salicylic acid, anaerobic induction, defense and stress, light, low temperature, MYB binding site (drought or light), and wound. (**B**) Potential protein–protein interaction network analysis. The orthologs of *A. thaliana* were used to predict potential interactions in the STRING database 12.0 [39]. The interaction network with a minimum required interaction score of 0.700 and FDR of 1% were shown and connected by gray lines. (**C**) Biological process (gene ontology, GO) enrichment. The *Y*-axis indicates different terms of GO biological process enrichment, and the *X*-axis indicates the corresponding gene count. The false discovery rate (FDR) and gene count are represented by green/blue blocks and circles of different sizes, respectively.

The 50 AP2/ERF genes that were consistently expressed (either up- or down-regulated) across all four stresses were selected for in-depth analysis (Figure 7). Most of these genes belonged to the ERF subfamily (approximately 64%) (Figure 7A). Among the 30 up-regulated genes, three, one, 24, and two genes belonged to the AP2, DREB (A5 group), ERF (15, eight, and one gene from B3, B4, and B5 groups, respectively), and RAV subfamilies, respectively. And the 20 down-regulated genes, four, eight, and eight genes belonged to the AP2, DREB subfamilies (one, five, and two genes from A4, A5, and A6 groups, respectively), and ERF subfamilies, respectively (two and six genes from B3 and B6 groups, respectively). Of these 50 genes, *GWHGAOPM041144* (B3 group, 54.76-fold), *GWHGAOPM046161* (B3 group, 31.03-fold), and *GWHGAOPM035982* (B4 group, 28.68-fold) were the most strongly up-regulated and *GWHGAOPM003956*, *GWHGAOPM023993*, and *GWHGAOPM023996* (all from the A3 group; 0.12-fold, 0.14-fold, and 0.16-fold, respectively) were the most strongly down-regulated. Additionally, there was a certain correlation between acid–aluminum, disease, and wound stresses, with the average correlation coefficients of 0.70 (*p* < 0.01), especially for disease and wound stresses (r = 0.91, *p* < 0.01) (Figure S4). This is likely because diseases often infect plants through tiny wounds.

Analysis of the *cis*-elements indicated that these genes contained multiple light-responsive elements, with an average of 10. And there were a relatively high number of other elements, such as abscisic acid, MeJA, anaerobic induction, and MYB binding site, averaging 3, 3, 2, and 1, respectively (Figure 7A). For example, the promoter of the *GWHGAOPM030794* gene contained 12 MeJA elements, and five genes (*GWHGAOPM012354*, *GWHGAOPM029486*, *GWHGAOPM005670*, *GWHGAOPM003956*, and *GWHGAOPM047836*) each had eight MeJA-responsive elements. Moreover, four genes (*GWHGAOPM045147*, *GWHGAOPM007570*, *GWHGAOPM030794*, *GWHGAOPM005670*) had ≥7 abscisic acid-responsive elements (Figure 7A). Furthermore, significant correlations (r ≥ 0.3) were observed among five pairs of *cis*-elements, including MYB bind site and salicylic acid (r = −0.8, *p* < 0.01), low temperature and gibberellin (r = 0.59, *p* < 0.05), as well as abscisic acid and gibberellin (r = 0.43, *p* < 0.05), MeJA (r = 0.40, *p* < 0.05), salicylic acid (r = 0.60, *p* < 0.05) (Figure S4). Integrating stress treatments with *cis*-element analysis revealed that MeJA elements show certain correlations with acid–aluminum (r = 0.40, *p* < 0.05), disease (r = 0.45, *p* < 0.01), and wound stress (r = 0.33, *p* < 0.05), while IAA *cis*-elements correlate with acid–aluminum stress (r = 0.46, *p* < 0.01) (Figure S4). Specifically, genes containing more MeJA elements might be more responsive to acid–aluminum, disease, and wound stress, whereas genes with more IAA elements might be more responsive to acid–aluminum stress. For example, the *GWHGAOPM030794* gene (containing 12 MeJA *cis*-elements) exhibited an average of 16-fold expression change under different stresses. *GWHGAOPM040096* and *GWHGAOPM009119* genes had three IAA *cis*-elements, and were up-regulated expressed.

The STRING database is a powerful tool for analyzing potential protein–protein interactions, providing a new way for predicting relationships of candidate proteins [39]. Since olives were not included in the database, 22 olive genes were detected and their orthologs in *A. thaliana* (including 13 genes) by the BLASTp tool with a stringent E-value cutoff of 1 × 10^−40^ and sequence similarity > 45% (Table S3), then the functional partners of orthologs were explored in STRING 12.0 [39]. Finally, nine *A. thaliana* orthologs were predicted to be associated with other proteins (Figure 7B, Table S3). Among them, three proteins AT5G47220 (orthologs of *GWHGAOPM041144*), AT3G23240 (orthologs of *GWHGAOPM012354*, *GWHGAOPM030794*, *GWHGAOPM007131*, *GWHGAOPM046161*), and AT4G36920 (orthologs of *GWHGAOPM002263*, *GWHGAOPM020378*) seemed to have more interactions with others, and interact with each other (Figure 7B, Table S3). AT5G47220 and AT3G23240 proteins had similar interaction networks with another seven proteins (ACO4, EIL1, EIL2, EIL3, EIL4, EIL5, and PDF1.2A). ACO4 encodes 1-aminocyclopropane-1-carboxylate oxidase, and is involved in the ethylene biosynthesis, while EIL proteins belong to the EIN3 family, and act as positive regulators in the ethylene response pathway. PDF1.2A confers broad-spectrum resistance to pathogens. AT4G36920 was predictively connected with the other four proteins, including ACO4, EIL1, PDF1.2A, and PLT3 (Figure 7B, Table S3), and PLT3 protein is a plasma membrane sugar-proton symporter, participating in sugar transport. Four proteins, AT2G28550 (orthologs of *GWHGAOPM045147* and *GWHGAOPM006104*), AT5G47230 (orthologs of *GWHGAOPM021434*), AT5G10510 (orthologs of *GWHGAOPM038678* and *GWHGAOPM002539*), and AT5G57390 (orthologs of *GWHGAOPM003646*) were also predicted to interact with ACO4, PDF1.2A, PLT3, and PLT3, respectively, and AT5G57390 also interacted with unknown protein F15M7.12 (Figure 7B, Table S3). Additionally, both AT1G13260 (orthologs of *GWHGAOPM010989*) and AT1G51120 (orthologs of *GWHGAOPM023375*) might interact with F25G13.130, an AP2/ERF (Soloist subfamily) protein. Biological process (gene ontology) enrichment analysis of these PPIs showed that pathways such as cellular process, response to organic substance, response to ethylene, cellular response to organic substance, ethylene-activated signaling pathway, post-embryonic plant organ development, and response to fatty acid were enriched (Figure 7C). This also suggests the important roles of AP2/ERF genes in plant morphogenesis and metabolism of organic substances and fatty acids.

## 4. Discussion

The AP2/ERF superfamily is a key class of transcription factors in plants, defined by a unique AP2 domain that confers specific DNA-binding ability. These factors play important roles in plant growth and development, stress adaptation, signal transduction, and the regulation of secondary metabolites, making them especially important for responses to various biotic and abiotic stresses [13,15,22,40]. The olive tree, an ancient tree species native to the Mediterranean region, has undergone over 6000 years of domestication and selection [19,20]. It has many desirable traits, such as drought tolerance and adaptability to infertile soils. It is cultivated in multiple countries across Europe, Asia, Africa, North America, South America, and Oceania, showing strong environmental adaptability [1,2,3,4].

In this study, we provided the first comprehensive genome-wide identification and analysis of the AP2/ERF superfamily in the widely cultivated olive variety ‘Arbequina’, with a total of 348 members obtained (Table 1 and Table S1). This number is substantially larger than those reported in diploid *A. thaliana* (122), rice (139), soybean (98), and maize (214), indicating lineage-specific expansion [9,10,11]. Following established classification criteria, the olive AP2/ERF genes were divided into four distinct families: AP2, ERF/DREB, RAV, and Soloist (Figure 1, Table 1 and Table S1). Among them, the ERF/DREB family was the largest (295 genes), further split into DREB and ERF subfamilies, each with six groups (A1–A6 and B1–B6, respectively). The notable expansion of this stress-associated superfamily [13,14,15] likely arose from frequent gene duplications, which were observed in 150 genes involving 61 tandem events and 229 genes involving 201 segmental events (Figure 2, Table S2). This is particularly reflected in the ERF/DREB family, which showed a total of nine types of motifs (Figure 3, Table S1). This may have been a key evolutionary driver enhancing olive’s adaptability to environmental pressures like drought and variable soil. In contrast, the conserved motifs and smaller family sizes of RAV and Soloist genes (Figure 3, Table S1) might reflect stronger functional constraints on their roles in fundamental developmental processes. Further comparative genomics with close relatives would help elucidate whether this uneven distribution is a shared trait within the Oleaceae family or a specific adaptation of olive.

The AP2/ERF genes are renowned for their pivotal role in integrating environmental and hormonal signals, a feature conserved across species [13,14,15]. In the present study, we employed promoter prediction to analyze the characteristics of the *cis*-elements of olive AP2/ERF genes (Figure 5), and the publicly available transcriptome data were also used to examine their response patterns to various environmental stresses (Figure 6). Promoter analysis revealed that olive AP2/ERF genes are enriched in *cis*-elements related to hormonal responses like ABA, IAA, GA, MeJA, and SA, and related to environmental factors like anaerobic induction, defense and stress responses, light, low temperature, and drought. Specifically, the average number of light- (mainly including G-Box, Box 4, GT1-motif, TCT-motif, and GATA-motif), MeJA- (CGTCA-motif and TGACG-motif), abscisic acid- (ABRE), anaerobic induction- (ARE) responsive elements was notably high compared to the other elements detected (Figure 5). Among them, the CGTCA-motif, TGACG-motif, ABRE, and ARE were also redundant in other plants like maize, *Brachypodium distachyon*, and *Prunus sibirica* [10,12,41], which seemed be shared by AP2/ERF genes. While they did not have many light-responsive elements, suggesting the photoresponsive pathway may have undergone specific amplification in olive. Analysis of the gene expression patterns also reflected that the AP2/ERF gene responds to various environmental changes, with 253, 251, 242, and 240 DEGs identified under acid–aluminum, wound, disease, and cold stresses, respectively (Figure 6). Additionally, most AP2/ERF genes had relatively low expression levels under normal conditions, unless induced by external stimuli. This is consistent with the expression characteristics reported for other species [12,41]. Notably, AP2/ERF members with high sequence similarity tended to have a similar *cis*-element composition and stress expression patterns in olive.

Fifty key AP2/ERF genes that were consistently up-regulated or down-regulated under multiple environmental stresses, as they may help plants adapt to complex natural conditions (Figure 7). Temperature is a key factor determining the global distribution of olive trees, and soils in traditional production areas are mostly neutral to weakly alkaline. Breeding of cold- and acid–aluminum-tolerant cultivars is necessary for expanding the olive industry. Strong disease resistance and wound tolerance are also important for stable yields [1,3]. The 50 AP2/ERF genes, mostly belonging to the ERF subfamily, had differential expression changes in response to cold, acid–aluminum, disease, and wound stresses. There existed significant correlations between MeJA *cis*-elements with acid–aluminum, disease, and wound stress, as well as IAA *cis*-elements with acid–aluminum stress (Figure 7 and Figure S4). These two hormones and their regulatory pathways in plant growth, development, and stress response have been confirmed in the functions of the AP2/ERF genes. MeJA can induce the expression of plant defense genes and is hydrolyzed to JA, which triggers the biosynthesis of various secondary metabolites [42,43]. While the homologs of *GWHGAOPM030794* and *GWHGAOPM012354* in *A. thaliana*, *AtERF1*, are known to participate in JA and ABA signaling pathways via *AtMYC2*, and to respond to light and *Botrytis cinerea* infection [12,44,45,46]. The *ORC1* in tobacco (*Nicotiana glauca*) and a basic helix-loop-helix (bHLH) factor mediate alkaloid biosynthesis via JA signal, and were post-translationally upregulated by MAPK proteins [47]. IAA mainly promotes the division and elongation of plant cells, further regulates the morphology and adversity. *OsERF096* in rice is found to respond to cold stress by regulating IAA accumulation and pathway [48]. Taken together, our analysis of AP2/ERF expression patterns and *cis*-elements, alongside functional studies of their homologs in other plants, revealed that these genes are involved in stress responses and are rarely induced by a single stress. Instead, they typically respond to changes in multiple environmental conditions via various pathways. Meanwhile, in addition to stress responses, AP2/ERF genes may also play important roles in plant morphogenesis, such as root and leaf development. By leveraging the STRING database 12.0 [39] of *Arabidopsis*, nine homologs of the candidate genes showed the potential interaction with other proteins (Figure 7B,C), which provides valuable predictive insights into the functional partnerships of AP2/ERF genes. It is crucial to note that this study is primarily a bioinformatics investigation. While the identified AP2/ERF genes display distinctive features in the olive tree, *cis*-elements provide strong regulatory hypotheses, and the PPI network offers valuable interaction candidates, these predictions require empirical validation. Future work must therefore pivot towards experimental validation. This includes functional studies using transgenic or gene editing approaches to confirm gene function under stress, in vitro assays such as yeast two-hybrid or co-immunoprecipitation to verify the predicted protein interactions, and electrophoretic mobility shift assays to confirm TF binding to predicted promoter elements. Furthermore, detailed analysis of the coding sequences and untranslated regions of these candidate genes could reveal important regulatory information, such as miRNA binding sites or regulatory SNPs, linking genetic variation to stress adaptation phenotypes.

## 5. Conclusions

In this study, we comprehensively identified and characterized the AP2/ERF superfamily in the olive genome, revealing 348 genes classified into the AP2, ERF (containing B1–B6 groups)/DREB (containing A1–A6 groups), RAV, and Soloist families. Through phylogenetic, chromosome distribution, structural, and promoter analysis, we established conserved patterns within each family, except for *cis*-elements within highly similar sequences. The stress response analysis revealed that 50 genes were consistently up-regulated (*n* = 30) or down-regulated (*n* = 20) by the treatments of acid–aluminum, cold, disease, and wound stresses, and nine homologs of which showed the potential interaction with other proteins. This study provides an important foundation for further exploring the roles and mechanisms of AP2/ERF genes and establishes a crucial gene source for advancing the research on olive stress adaptation. While this systematic bioinformatics analysis provides a foundational gene inventory and valuable hypotheses regarding stress-responsive candidates, it is primarily predictive and requires more experimental validation. The statistical re-analysis, enrichment testing, and orthogonal validation will elevate mechanistic confidence and translational relevance. Future work should prioritize functional characterization of the highlighted core genes through transgenic approaches and molecular studies.

## Figures and Tables

**Table 1:** Summary of AP2/ERF superfamily in olive tree.

Family ^1^	Subfamily	Groups	No. of Genes	Length of Amino Acids	Molecular Weight (MW)/kDa	Isoelectric Point (pI)	Instability Index
AP2	-	-	46	447	50.1	7.27	49.1
ERF/DREB	DREB	A1	6	210	23.7	7.20	52.5
		A2	11	288	32.3	5.41	41.6
		A3	2	302	32.2	7.97	59.6
		A4	33	221	24.1	6.32	56.0
		A5	29	179	19.8	7.05	52.3
		A6	22	330	36.8	7.40	51.5
		Total (DREB)	103	241	26.6	6.74	52.3
	ERF	B1	26	247	27.2	7.21	55.3
		B2	8	300	33.3	5.76	39.7
		B3	68	189	21.4	6.94	53.4
		B4	28	286	31.7	8.20	56.3
		B5	16	318	35.8	5.57	44.6
		B6	46	254	28.6	6.90	52.6
		Total (ERF)	192	242	27.1	6.99	52.6
	Total (ERF/DREB)	-	295	241	26.9	6.90	52.5
RAV	-	-	4	350	39.4	7.33	40.9
Soloist	-	-	3	215	24.4	9.84	46.5
Total (AP2/ERF)	-	-	348	270	30.1	6.98	51.9

^1^ AP2, APETALA2; ERF, Ethylene response factor; DREB, Dehydration-responsive element binding proteins; RAV, Related to ABI3/VP1.

## Data Availability

The original contributions presented in this study are included in the article and Supplementary Material. Further inquiries can be directed to the corresponding authors.

## Supplementary Materials

The following supporting information can be downloaded at: https://www.mdpi.com/article/10.3390/cimb48020183/s1.

## Abbreviations

The following abbreviations are used in this manuscript:

AP2	APETALA2
ERF	Ethylene response factor
DREB	Dehydration-responsive element binding proteins
DRE	Dehydration response element
RAV	Related to ABI3/VP1
CRT	C-repeat
ABA	Abscisic acid
ERE	Ethylene response element
HRPE	Hypoxia-responsive promoter element
MV	Methyl viologen
GA	Gibberellin
BR	Brassinosteroid
JA	Jasmonic acid
IAA	Indole-3-acetic acid
CDD	Conserved Domains Database
MW	Molecular weight
pI	Isoelectric point
TAIR	The *Arabidopsis* Information Resource
TPM	Transcripts per million
DEGs	Differentially expressed genes
GO	Gene ontology
HMM	Hidden Markov Model
UTR	Untranslated regions
MeJA	Methyl jasmonate
SA	Salicylic acid
