# Supplementary material for: Comprehensive Analysis of the AP2/ERF Superfamily Identifies Key Genes Related to Various Stress Responses in Olive Tree (Olea europaea L.)
Source: Curr Issues Mol Biol. 2026 Feb 5;48(2):183. doi: 10.3390/cimb48020183 (PMC12939568; doi:10.3390/cimb48020183)
Supplement: Supplementary file 1 [file cimb-48-00183-s001.zip › Supplementary figures and tables.pdf]

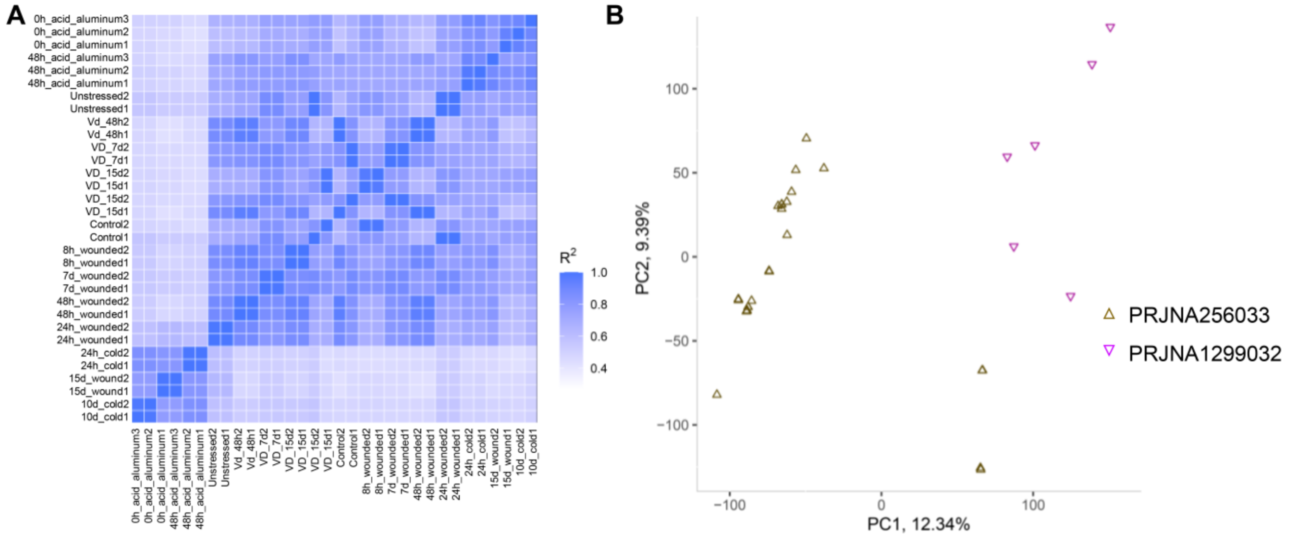

Figure S1. Pearson correlation (**A**) and PCA analysis (**B**) of different transcriptome profiles.

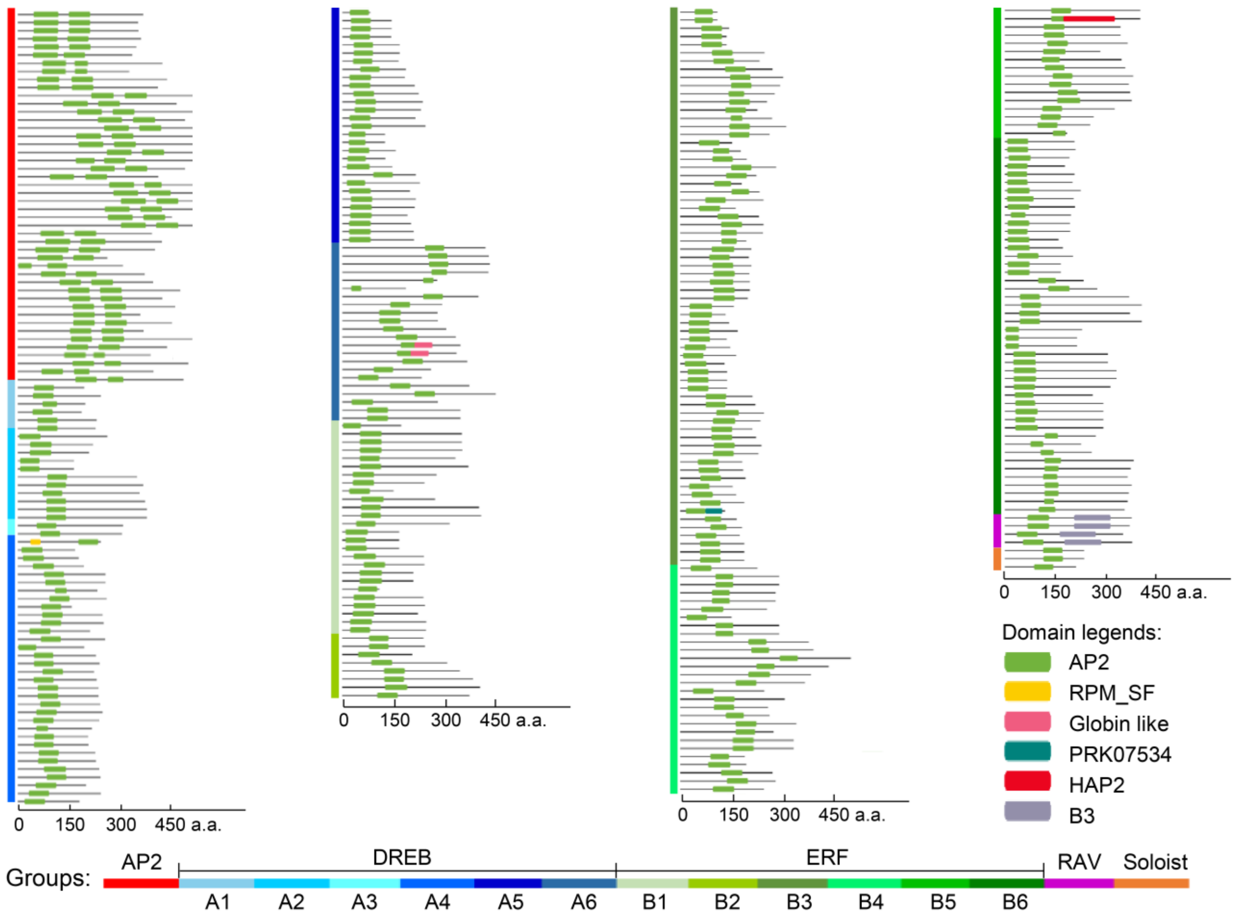

Figure S2. Detailed diagrams of conserved domains of AP2/ERF-encoded proteins. The domains were represented by boxes of different colors. Different groups are presented in different color blocks on the left, and the gene names of corresponding proteins are listed in Table S1.

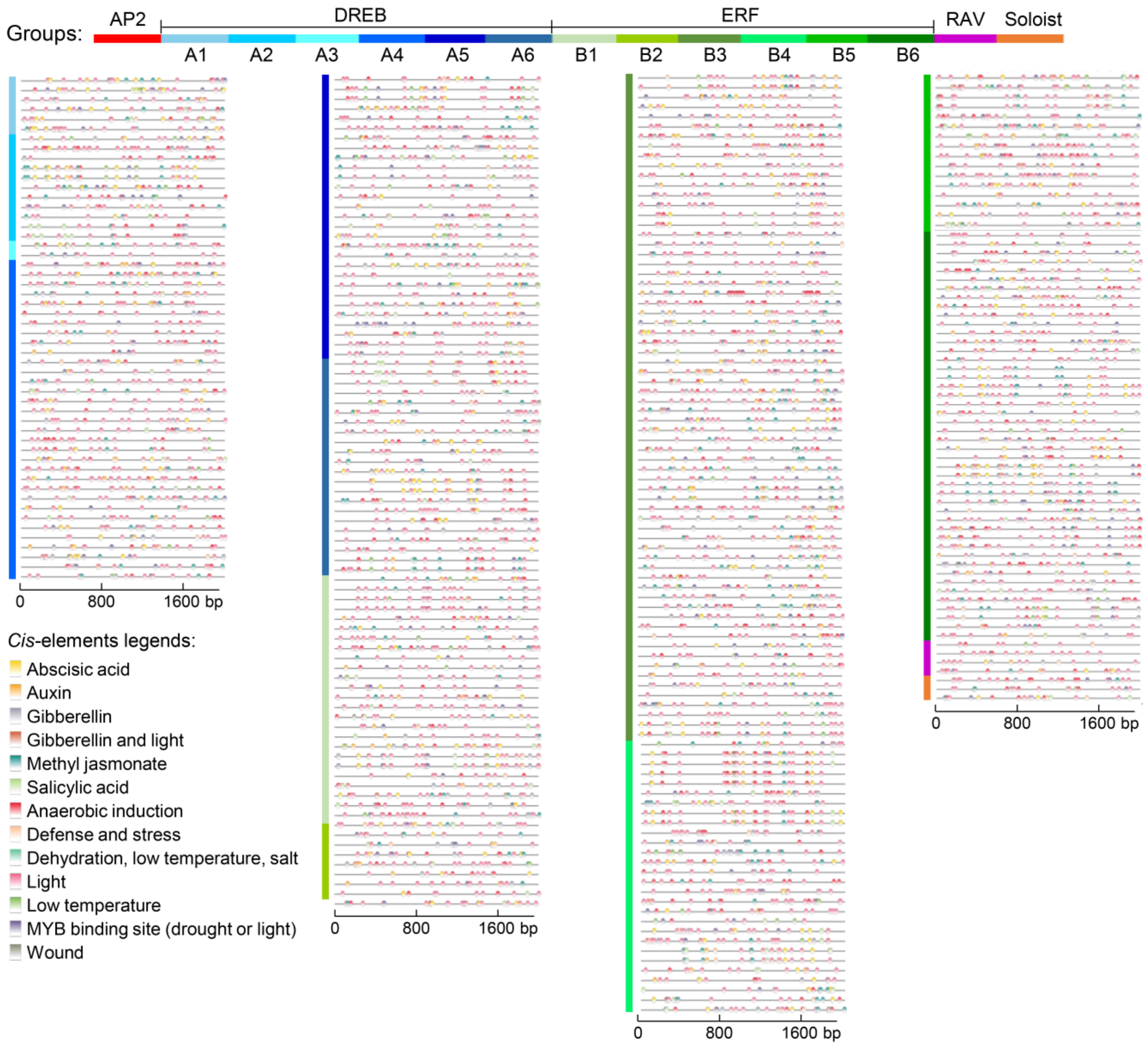

Figure S3. *Cis*-elements predicted in the promoters of AP2/ERF genes (excluding the AP2 subfamily). The 2000-bp sequence upstream of the 5' end of the open reading frame of each gene was analyzed to predict these cis-elements, which are represented by circles of different colors. Different groups are presented in different color blocks on the left, and the corresponding gene names are listed in Table S1.

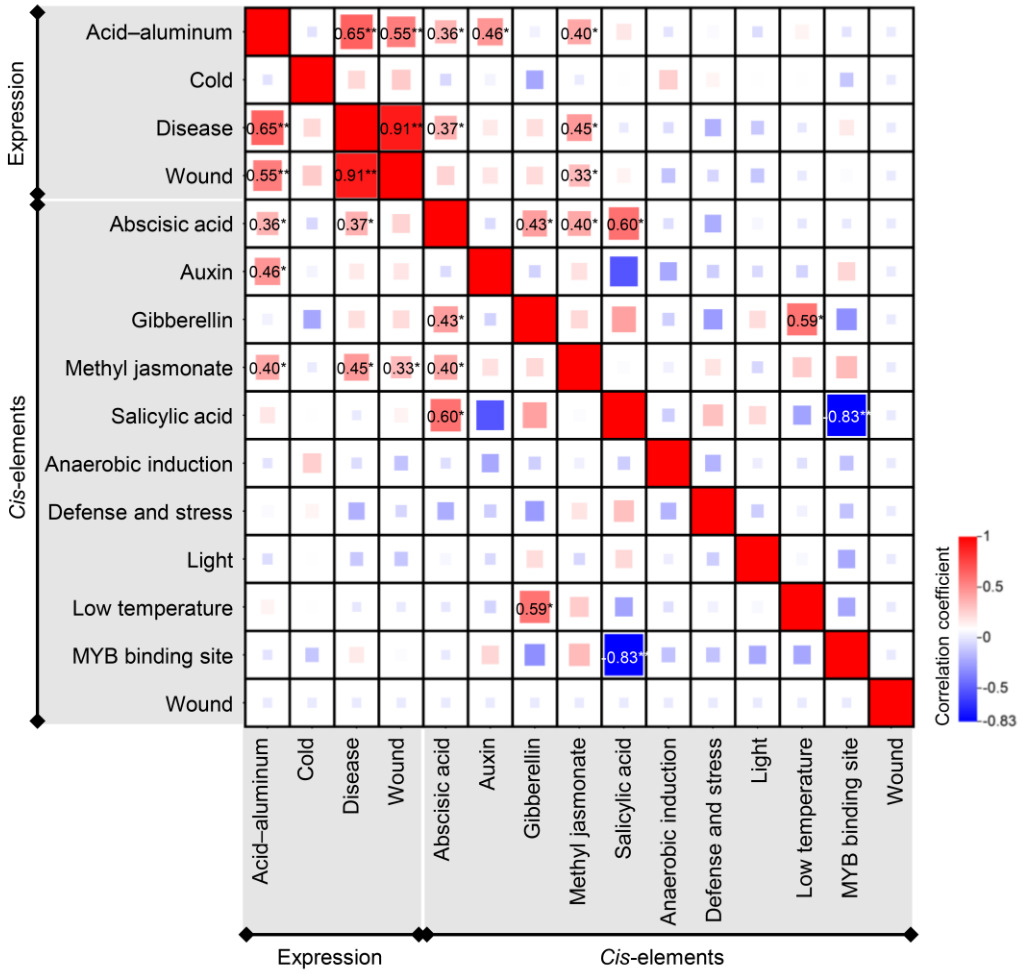

Figure S4. Pearson correlation analysis between expression of stress response and *cis*-elements. The correlation coefficients at the significant levels of $p < 0.05$ (*) and $p < 0.01$(**) were displayed.

Table S1. The gene information of AP2/ERF superfamily in olive tree.

Family	Subfamily	Groups	Gene ID	Subcellular location[1]	Location/Mb	Domain organization	Main motifs[2]
AP2	-	-	GWHGAOPM047585	Nuc	Chr23: 11.64	AP2×2	1, 2, 3, 4, 5, 6, 7
			GWHGAOPM047562	Nuc	Chr23: 11.4	AP2×2	1, 2, 3, 4, 5, 6, 7
			GWHGAOPM024554	Nuc	Chr12: 9.22	AP2×2	1, 2, 3, 4, 5, 6, 7
			GWHGAOPM019285	Cyt/Nuc	Chr10: 40.64	AP2×2	1, 2, 3, 4, 5, 6, 7
			GWHGAOPM023303	Cyt/Nuc	Chr11: 42.95	AP2×2	1, 2, 3, 4, 5, 6, 7
			GWHGAOPM048521	Nuc	Chr23: 33.1	AP2×2	1, 2, 3, 5, 6, 7
			GWHGAOPM027304	Nuc	Chr13: 9.99	AP2×2	1, 3, 4
			GWHGAOPM039967	Nuc	Chr19: 7.1	AP2×2	1, 3, 4
			GWHGAOPM002356	Cyt/Nuc	Chr02: 3.39	AP2×2	1, 3, 4, 6, 7
			GWHGAOPM004882	Cyt/Nuc	Chr03: 9.81	AP2×2	1, 3, 4, 5, 6, 7
			GWHGAOPM028491	Nuc	Chr13: 34.06	AP2×2	1, 3, 4, 5, 6, 7
			GWHGAOPM003646	Nuc	Chr02: 36.45	AP2×2	1, 2, 3, 4, 5, 6, 7
			GWHGAOPM025248	Nuc	Chr12: 19.43	AP2×2	1, 2, 3, 4, 5, 6, 7
			GWHGAOPM038678	Nuc	Chr18: 31.62	AP2×2	1, 2, 3, 4, 6, 7
			GWHGAOPM041320	Nuc	Chr19: 32.27	AP2×2	1, 2, 3, 4, 5, 6, 7
			GWHGAOPM006192	Nuc	Chr04: 3.02	AP2×2	1, 2, 3, 4, 5, 6, 7
			GWHGAOPM049162	Nuc	Contig001255 : 0.14	AP2×2	1, 2, 3, 4, 5, 6, 7
			GWHGAOPM047999	Nuc	Chr23: 19.05	AP2×2	1, 2, 3, 4, 5, 6, 7
			GWHGAOPM001268	Nuc	Chr01: 19.65	AP2×2	1, 3, 4, 5, 6, 7
			GWHGAOPM002539	Nuc	Chr02: 7.46	AP2×2	1, 3, 4, 6, 7
			GWHGAOPM007277	Nuc	Chr04: 25.95	AP2×2	1, 3, 4, 5, 6, 7
			GWHGAOPM039086	Nuc	Chr18: 38.97	AP2×2	1, 2, 3, 4, 6
			GWHGAOPM030456	Nuc	Chr14: 23.58	AP2×2	1, 2, 3, 4, 5, 6, 7
			GWHGAOPM012908	Nuc	Chr07: 15.79	AP2×2	1, 2, 3, 4, 5, 6, 7
			GWHGAOPM046315	Nuc	Chr22: 24.88	AP2×2	1, 2, 3, 4, 5, 6, 7
			GWHGAOPM035843	Nuc	Chr17: 10.2	AP2×2	1, 2, 3, 4, 5,

							6, 7
			GWHGAOPM020470	Nuc	Chr10: 56.89	AP2×2	1, 2, 3, 4, 5, 6, 7
			GWHGAOPM039423	Nuc	Chr18: 43.94	AP2×2	1, 3, 4, 5, 6, 7
			GWHGAOPM011359	Nuc	Chr06: 41.94	AP2×2	1, 3, 4, 5, 6, 7
			GWHGAOPM020124	Nuc	Chr10: 51.65	AP2×2	1, 3, 4, 5, 6, 7
			GWHGAOPM036840	Nuc	Chr17: 32.4	AP2×2	1, 2, 3, 4, 6, 7
			GWHGAOPM039422	Cyt/Nuc	Chr18: 43.94	AP2×2	3, 4, 5, 6, 7
			GWHGAOPM013152	Nuc	Chr07: 22.34	AP2×2	1, 3, 4, 5, 6, 7
			GWHGAOPM002283	Cyt	Chr02: 1.99	AP2×2	1, 3, 4, 5, 6, 7
			GWHGAOPM042560	Nuc	Chr20: 7.2	AP2×2	1, 3, 4, 5, 6, 7
			GWHGAOPM002263	Cyt	Chr02: 1.63	AP2×2	1, 3, 4, 5, 6, 7
			GWHGAOPM036979	Nuc	Chr17: 35.77	AP2×2	1, 3, 4, 5, 6, 7
			GWHGAOPM024049	Cyt	Chr12: 0.73	AP2×2	1, 4, 5, 6, 7
			GWHGAOPM024042	Nuc	Chr12: 0.59	AP2×2	1, 3, 4, 5, 6, 7
			GWHGAOPM039190	Nuc	Chr18: 40.87	AP2×2	1, 3, 4, 5, 6, 7
			GWHGAOPM020378	Nuc	Chr10: 55.42	AP2×2	1, 3, 4, 5, 6, 7
			GWHGAOPM045147	Nuc	Chr22: 1.16	AP2×2	1, 3, 4, 5, 6, 7
			GWHGAOPM006104	Nuc	Chr04: 1.59	AP2×2	1, 4, 6, 7
			GWHGAOPM000634	Nuc	Chr01: 9.22	AP2×2	1, 4, 5, 6, 7
			GWHGAOPM042845	Nuc	Chr20: 15.83	AP2×2	1, 3, 4, 6, 7
			GWHGAOPM042851	Nuc	Chr20: 16.06	AP2×2	1, 3, 4, 6, 7
ERF/DREB	DREB	A1	GWHGAOPM022567	Nuc	Chr11: 24.32	AP2×1	2, 5, 6, 7, 8, 9
			GWHGAOPM043453	Cyt	Chr20: 28.6	AP2×1	2, 5, 6, 7, 8, 9
			GWHGAOPM021268	Nuc	Chr11: 4.34	AP2×1	2, 5, 6, 9
			GWHGAOPM041083	Nuc	Chr19: 26.01	AP2×1	2, 5, 6, 7, 8
			GWHGAOPM051657	Nuc	Contig01204: 0.06	AP2×1	2, 5, 6, 7, 8
			GWHGAOPM041084	Cyt/Nuc	Chr19: 26.02	AP2×1	2, 5, 6, 7, 8
		A2	GWHGAOPM016547	Nuc	Chr09: 17.81	AP2×1	5, 6, 7
			GWHGAOPM013785	Cyt/Nuc	Chr07: 34.01	AP2×1	2, 5, 6, 7
			GWHGAOPM013784	Nuc	Chr07: 34	AP2×1	5, 6, 7
			GWHGAOPM049871	Nuc	Contig001263: 0.1	AP2×1	2, 5, 6, 7
			GWHGAOPM053360	Nuc	Contig01249: 0.1	AP2×1	2, 5, 6, 7
			GWHGAOPM041518	Nuc	Chr19: 39.08	AP2×1	2, 5, 6, 7, 9
			GWHGAOPM001944	Nuc	Chr01: 35.64	AP2×1	2, 5, 6, 7
			GWHGAOPM000005	Nuc	Chr01: 0.09	AP2×1	2, 5, 6, 7
			GWHGAOPM024190	Nuc	Chr12: 2.57	AP2×1	2, 5, 6, 7
			GWHGAOPM000775	Nuc	Chr01: 11.85	AP2×1	2, 5, 6, 7
			GWHGAOPM000784	Nuc	Chr01: 12.02	AP2×1	2, 5, 6, 7
		A3	GWHGAOPM001940	Nuc	Chr01: 35.57	AP2×1	2, 5, 6, 7

	GWHGAOPM025866	Nuc	Chr12: 32.36	AP2×1	2, 5, 6, 7
A4	GWHGAOPM011467	Chl/Cyt	Chr06: 44.19	AP2×1	5, 6, 7
	GWHGAOPM008145	Nuc	Chr05: 6.33	AP2×1	2, 5, 6, 7, 8
	GWHGAOPM029232	Nuc	Chr14: 0.46	AP2×1	2, 5, 6, 7, 8
	GWHGAOPM030980	Nuc	Chr15: 0.32	AP2×1	2, 5, 6, 7, 8
	GWHGAOPM008766	Nuc	Chr05: 19.9	AP2×1	2, 5, 6, 7, 8
	GWHGAOPM034407	Nuc	Chr16: 24.87	AP2×1	1, 2, 5, 6, 7, 8
	GWHGAOPM005465	Nuc	Chr03: 26.02	AP2×1	2, 6, 7, 8
	GWHGAOPM031732	Nuc	Chr15: 13.37	AP2×1	2, 5, 6, 7, 8
	GWHGAOPM015937	Nuc	Chr09: 2.59	AP2×1	2, 5, 6, 7, 8
	GWHGAOPM004776	Nuc	Chr03: 7.8	AP2×1	2, 5, 6, 7, 8
	GWHGAOPM033049	Nuc	Chr15: 40.67	AP2×1	2, 5, 6, 7, 8
	GWHGAOPM037820	Nuc	Chr18: 9.94	AP2×1	2, 5, 6, 7, 8
	GWHGAOPM011827	Nuc	Chr06: 49.85	AP2×1	2, 5, 6, 7, 8
	GWHGAOPM046433	Nuc	Chr22: 27.66	AP2×1	6, 7, 8
	GWHGAOPM007797	Nuc	Chr04: 36.22	AP2×1	2, 5, 6, 7
	GWHGAOPM007790	Nuc	Chr04: 36.12	AP2×1	2, 5, 6, 7, 8
	GWHGAOPM014861	Nuc	Chr08: 11.61	AP2×1	2, 5, 6, 7, 8
	GWHGAOPM041280	Nuc	Chr19: 31.13	AP2×1	2, 5, 6, 7, 8
	GWHGAOPM035798	Nuc	Chr17: 9.52	AP2×1	2, 5, 6, 7, 8, 9
	GWHGAOPM035793	Nuc	Chr17: 9.39	AP2×1	2, 5, 6, 7, 8, 9
	GWHGAOPM004047	Nuc	Chr02: 42.26	AP2×1	2, 5, 6, 7, 8
	GWHGAOPM028960	Nuc	Chr13: 46.51	AP2×1	2, 5, 6, 7, 8
	GWHGAOPM016165	Nuc	Chr09: 7.32	AP2×1	2, 5, 6, 7, 8
	GWHGAOPM050029	Nuc	Contig001267: 0.03	AP2×1	2, 5, 6
	GWHGAOPM001835	Nuc	Chr01: 33.13	AP2×1	2, 6, 7, 8, 9
	GWHGAOPM026038	Nuc	Chr12: 35.81	AP2×1	5, 6, 7, 8, 9
	GWHGAOPM011468	Cyt/Nuc	Chr06: 44.2	AP2×1	5, 6, 7, 8, 9
	GWHGAOPM014413	Nuc	Chr08: 2.02	AP2×1	5, 6, 7, 8, 9
	GWHGAOPM007851	Nuc	Chr04: 36.99	AP2×1	5, 6, 7, 8, 9
	GWHGAOPM041085	Nuc	Chr19: 26.05	AP2×1	5, 6, 7, 8, 9
	GWHGAOPM053371	Nuc	Contig01249: 0.08	AP2×1	5, 6, 7, 8
	GWHGAOPM017031	Cyt/Nuc	Chr09: 28.96	AP2×1	2, 5, 6, 7, 8, 9
	GWHGAOPM042652	Nuc	Chr20: 9.49	AP2×1	2, 5, 6, 7, 8, 9
A5	GWHGAOPM010521	Nuc	Chr06: 24.23	AP2×1	2, 5, 6, 7
	GWHGAOPM050591	Cyt/Nuc	Contig001289: 0.14	AP2×1	2, 5, 6, 7, 8
	GWHGAOPM052831	Cyt/Nuc	Contig01234: 0.07	AP2×1	2, 5, 6, 7, 8
	GWHGAOPM008233	Nuc	Chr05: 8.73	AP2×1	2, 5, 6, 7, 8
	GWHGAOPM039188	Nuc	Chr18: 40.8	AP2×1	2, 5, 6, 7
	GWHGAOPM020382	Nuc	Chr10: 55.56	AP2×1	2, 5, 6, 7

	GWHGAOPM013232	Nuc	Chr07: 24.15	AP2×1	2, 5, 6, 7
	GWHGAOPM030136	Nuc	Chr14: 15.1	AP2×1	2, 5, 6, 7
	GWHGAOPM003957	Cyt/Nuc	Chr02: 41.03	AP2×1	2, 5, 6, 7, 8
	GWHGAOPM048197	Cyt/Nuc	Chr23: 23.18	AP2×1	2, 5, 6, 7, 8
	GWHGAOPM048114	Nuc	Chr23: 21.39	AP2×1	2, 5, 6, 7, 8
	GWHGAOPM018920	Nuc	Chr10: 35.12	AP2×1	2, 5, 6, 7, 8
	GWHGAOPM018921	Nuc	Chr10: 35.13	AP2×1	2, 5, 6, 7, 8
	GWHGAOPM014206	Nuc	Chr07: 40.62	AP2×1	2, 5, 6, 7, 8
	GWHGAOPM029369	Cyt/Nuc	Chr14: 2.47	AP2×1	2, 5, 6, 7, 8
	GWHGAOPM012180	Nuc	Chr06: 54.62	AP2×1	2, 5, 6
	GWHGAOPM012187	Nuc	Chr06: 54.76	AP2×1	2, 5, 6
	GWHGAOPM008141	Nuc	Chr05: 6.28	AP2×1	2, 5, 6, 7
	GWHGAOPM008142	Cyt/Nuc	Chr05: 6.28	AP2×1	2, 6, 7, 8
	GWHGAOPM018819	Cyt/Nuc	Chr10: 33.21	AP2×1	2, 5, 6, 7
	GWHGAOPM025463	Cyt/Nuc	Chr12: 23.15	AP2×1	2, 5, 6, 7
	GWHGAOPM003956	Cyt/Nuc	Chr02: 41	AP2×1	2, 5, 6, 7, 8
	GWHGAOPM007753	Cyt/Nuc	Chr04: 35.56	AP2×1	2, 5, 6, 7, 8
	GWHGAOPM003000	Cyt/Nuc	Chr02: 22.57	AP2×1	2, 5, 6, 7, 8
	GWHGAOPM028110	Cyt/Nuc	Chr13: 24.08	AP2×1	2, 5, 6, 7, 8
	GWHGAOPM014774	Cyt/Nuc	Chr08: 9.72	AP2×1	2, 5, 6, 7, 8
	GWHGAOPM046464	Cyt/Nuc	Chr22: 28.4	AP2×1	2, 5, 6, 7, 8
	GWHGAOPM023996	Cyt/Nuc	Chr11: 56.92	AP2×1	2, 5, 6, 7, 8
	GWHGAOPM023993	Cyt/Nuc	Chr11: 56.84	AP2×1	2, 5, 6, 7, 8
A6	GWHGAOPM051880	Nuc	Contig01207: 0.11	AP2×1	2, 5, 6, 7
	GWHGAOPM030723	Nuc	Chr14: 30.33	AP2×1	2, 5, 6, 7
	GWHGAOPM050698	Nuc	Contig001290: 0.08	AP2×1	2, 5, 6, 7
	GWHGAOPM012435	Cyt/Nuc	Chr07: 4.17	AP2×1	2, 5, 6, 7
	GWHGAOPM006970	Cyt/Nuc	Chr04: 16.48	AP2×1	2, 5
	GWHGAOPM047342	Nuc	Chr23: 7.55	AP2×1	2, 5
	GWHGAOPM006966	Nuc	Chr04: 16.39	AP2×1	2, 5, 6, 7
	GWHGAOPM006975	Nuc	Chr04: 16.56	AP2×1	2, 5, 6, 7
	GWHGAOPM021026	Nuc	Chr11: 1.14	AP2×1	2, 5, 6, 7
	GWHGAOPM041692	Nuc	Chr19: 42.72	AP2×1	2, 5, 6, 7
	GWHGAOPM007906	Nuc	Chr04: 37.74	AP2×1	2, 5, 6, 7
	GWHGAOPM029550	Nuc	Chr14: 5.24	AP2×1	2, 5, 6, 7
	GWHGAOPM014026	Nuc	Chr07: 37.78	AP2×1	2, 5, 6, 7
	GWHGAOPM014030	Nuc	Chr07: 37.87	AP2×1	2, 5, 6, 7
	GWHGAOPM038759	Nuc	Chr18: 33.26	AP2×1	2, 4, 5, 6, 7
	GWHGAOPM020745	Nuc	Chr10: 61.39	AP2×1	2, 5, 6, 7
	GWHGAOPM037199	Nuc	Chr17: 40.12	AP2×1	2, 5, 6, 7
	GWHGAOPM019620	Cyt/Nuc	Chr10: 44.86	AP2×1	2, 5, 6, 7
	GWHGAOPM043800	Nuc	Chr21: 4.4	AP2×1	2, 5, 6, 7

		GWHGAOPM033187	Nuc	Chr15: 42.75	AP2×1	4, 5, 6, 7, 9
		GWHGAOPM001250	Nuc	Chr01: 19.43	AP2×1	5, 6, 7
		GWHGAOPM052612	Nuc	Contig01228: 0.02	AP2×1	5, 6, 7
ERF	B1	GWHGAOPM046941	Cyt	Chr23: 1.81	AP2×1	6, 7
		GWHGAOPM003831	Nuc	Chr02: 39.12	AP2×1	2, 5, 6, 7
		GWHGAOPM003821	Nuc	Chr02: 38.77	AP2×1	2, 5, 6, 7
		GWHGAOPM003835	Nuc	Chr02: 39.19	AP2×1	2, 5, 6, 7
		GWHGAOPM035573	Nuc	Chr17: 5.91	AP2×1	2, 5, 6, 7
		GWHGAOPM006803	Nuc	Chr04: 13.56	AP2×1	2, 5, 6, 7
		GWHGAOPM010543	Nuc	Chr06: 24.93	AP2×1	2, 5, 6, 7
		GWHGAOPM026423	Nuc	Chr12: 46.54	AP2×1	2, 5, 6, 7
		GWHGAOPM046762	Nuc	Chr22: 34.76	AP2×1	2, 5, 6
		GWHGAOPM036720	Nuc	Chr17: 29.58	AP2×1	2, 5, 6, 7
		GWHGAOPM010688	Nuc	Chr06: 28.89	AP2×1	2, 5, 6, 7
		GWHGAOPM015249	Nuc	Chr08: 21.4	AP2×1	2, 5, 6, 7
		GWHGAOPM022265	Nuc	Chr11: 19.53	AP2×1	2, 5, 6, 7
		GWHGAOPM010435	Nuc	Chr06: 21.15	AP2×1	2, 5, 6, 7
		GWHGAOPM027125	Nuc	Chr13: 7.29	AP2×1	2, 5, 6, 7
		GWHGAOPM040108	Nuc	Chr19: 9.13	AP2×1	2, 5, 6, 7
		GWHGAOPM026822	Nuc	Chr13: 3.38	AP2×1	2, 5, 6, 7
		GWHGAOPM039870	Nuc	Chr19: 4.88	AP2×1	2, 5, 6, 7
		GWHGAOPM051697	Nuc	Contig01205: 0.13	AP2×1	2, 5, 6, 7
		GWHGAOPM050392	Nuc	Contig001282 : 0.09	AP2×1	2, 5, 6, 7
		GWHGAOPM025172	Nuc	Chr12: 18.16	AP2×1	2, 5, 6, 7
		GWHGAOPM044695	Nuc	Chr21: 21.81	AP2×1	2, 5, 6, 7
		GWHGAOPM019488	Nuc	Chr10: 43.19	AP2×1	2, 5, 6, 7
		GWHGAOPM009507	Nuc	Chr06: 1.72	AP2×1	2, 5, 6, 7
		GWHGAOPM040107	Nuc	Chr19: 9.12	AP2×1	2, 5, 6, 7
		GWHGAOPM027126	Nuc	Chr13: 7.3	AP2×1	2, 5, 6, 7
	B2	GWHGAOPM045804	Nuc	Chr22: 10.41	AP2×1	2, 5, 6, 7
		GWHGAOPM006858	Nuc	Chr04: 14.36	AP2×1	2, 5, 6, 7
		GWHGAOPM022681	Nuc	Chr11: 26.42	AP2×1	2, 5, 6, 7
		GWHGAOPM024963	Nuc	Chr12: 14.98	AP2×1	2, 5, 6, 7, 8
		GWHGAOPM024967	Nuc	Chr12: 15.02	AP2×1	2, 5, 6, 7
		GWHGAOPM037962	Nuc	Chr18: 12.77	AP2×1	2, 5, 6, 7
		GWHGAOPM017938	Nuc	Chr10: 15.85	AP2×1	2, 5, 6, 7
		GWHGAOPM004068	Nuc	Chr02: 42.62	AP2×1	5, 6, 7
	B3	GWHGAOPM025985	Cyt	Chr12: 35	AP2×1	5, 6, 7
		GWHGAOPM025964	Cyt	Chr12: 34.64	AP2×1	5, 6, 7
		GWHGAOPM025969	Cyt/Nuc	Chr12: 34.7	AP2×1	2, 5, 6, 7
		GWHGAOPM025971	Nuc	Chr12: 34.73	AP2×1	2, 5, 6, 7

GWHGAOPM025988	Nuc	Chr12: 35.02	AP2×1	2, 5, 6, 7
GWHGAOPM024336	Nuc	Chr12: 5.54	AP2×1	2, 5, 6, 7
GWHGAOPM000848	Nuc	Chr01: 12.85	AP2×1	2, 5, 6, 7
GWHGAOPM042013	Nuc	Chr19: 47.94	AP2×1	2, 5, 6, 7, 9
GWHGAOPM021434	Nuc	Chr11: 6.51	AP2×1	2, 5, 6, 7, 9
GWHGAOPM007569	Nuc	Chr04: 31.82	AP2×1	2, 5, 6, 7, 9
GWHGAOPM046602	Nuc	Chr22: 32.24	AP2×1	6, 7, 9
GWHGAOPM028250	Nuc	Chr13: 27.98	AP2×1	2, 5, 6, 7, 9
GWHGAOPM028255	Nuc	Chr13: 28.38	AP2×1	2, 5, 6, 7, 9
GWHGAOPM028257	Cyt/Nuc	Chr13: 28.55	AP2×1	2, 6, 7, 9, 10
GWHGAOPM028254	Nuc	Chr13: 28.28	AP2×1	2, 5, 6, 7, 9
GWHGAOPM028256	Nuc	Chr13: 28.49	AP2×1	2, 5, 6, 7, 9
GWHGAOPM028252	Nuc	Chr13: 28.14	AP2×1	2, 5, 6, 7
GWHGAOPM028248	Nuc	Chr13: 27.94	AP2×1	2, 6, 7, 9
GWHGAOPM028261	Nuc	Chr13: 28.77	AP2×1	2, 3, 5, 6, 7, 9
GWHGAOPM046601	Nuc	Chr22: 32.24	AP2×1	2, 5, 6, 7, 9
GWHGAOPM021433	Nuc	Chr11: 6.48	AP2×1	2, 5, 6, 7, 9
GWHGAOPM024337	Nuc	Chr12: 5.55	AP2×1	5, 6, 7, 9
GWHGAOPM000849	Nuc	Chr01: 12.85	AP2×1	2, 5, 6, 7, 9
GWHGAOPM046931	Nuc	Chr23: 1.67	AP2×1	2, 5, 6, 7, 9
GWHGAOPM040096	Nuc	Chr19: 8.96	AP2×1	2, 5, 6, 7
GWHGAOPM042017	Nuc	Chr19: 48	AP2×1	2, 5, 6, 7
GWHGAOPM021435	Nuc	Chr11: 6.53	AP2×1	2, 5, 6, 7
GWHGAOPM024335	Nuc	Chr12: 5.52	AP2×1	2, 6, 7
GWHGAOPM046600	Nuc	Chr22: 32.2	AP2×1	2, 5, 6
GWHGAOPM007570	Nuc	Chr04: 31.84	AP2×1	2, 5, 6, 7
GWHGAOPM028262	Nuc	Chr13: 28.84	AP2×1	2, 5, 6, 7
GWHGAOPM041144	Nuc	Chr19: 27.09	AP2×1	2, 5, 6, 7
GWHGAOPM008743	Nuc	Chr05: 19.46	AP2×1	2, 5, 6, 7
GWHGAOPM008747	Nuc	Chr05: 19.53	AP2×1	2, 5, 6, 7
GWHGAOPM008748	Nuc	Chr05: 19.64	AP2×1	2, 5, 6, 7
GWHGAOPM008744	Cyt/Nuc	Chr05: 19.48	AP2×1	2, 5, 6, 7
GWHGAOPM046164	Nuc	Chr22: 19.56	AP2×1	2, 5, 6, 7, 9
GWHGAOPM007130	Nuc	Chr04: 20.83	AP2×1	2, 5, 6, 7, 9
GWHGAOPM030797	Nuc	Chr14: 32.15	AP2×1	2, 5, 6, 7, 9
GWHGAOPM009120	Nuc	Chr05: 25.01	AP2×1	2, 5, 6, 7, 9
GWHGAOPM034926	Nuc	Chr16: 32.99	AP2×1	2, 5, 6, 7, 9
GWHGAOPM034924	Nuc	Chr16: 32.97	AP2×1	2, 5, 6, 7, 9
GWHGAOPM009118	Nuc	Chr05: 24.99	AP2×1	5, 6, 7, 9
GWHGAOPM009119	Nuc	Chr05: 25	AP2×1	2, 5, 6, 7, 9
GWHGAOPM007132	Nuc	Chr04: 20.91	AP2×1	2, 5, 6, 7, 9
GWHGAOPM030798	Nuc	Chr14: 32.15	AP2×1	2, 5, 6, 7, 9
GWHGAOPM012353	Nuc	Chr07: 2.9	AP2×1	2, 5, 6, 7, 9
GWHGAOPM040299	Nuc	Chr19: 12.06	AP2×1	2, 5, 6, 7, 9,

					10
	GWHGAOPM027700	Nuc	Chr13: 16.95	AP2×1	2, 5, 6, 7, 9, 10
	GWHGAOPM012354	Nuc	Chr07: 2.93	AP2×1	2, 5, 6, 7, 9, 10
	GWHGAOPM030794	Nuc	Chr14: 32.04	AP2×1	2, 5, 6, 7, 9, 10
	GWHGAOPM007131	Nuc	Chr04: 20.88	AP2×1	2, 5, 6, 7, 9, 10
	GWHGAOPM046161	Nuc	Chr22: 19.46	AP2×1	2, 5, 6, 7, 9, 10
	GWHGAOPM034923	Nuc	Chr16: 32.96	AP2×1	2, 5, 6, 7, 9, 10
	GWHGAOPM022794	Nuc	Chr11: 28.92	AP2×1	2, 5, 6, 7, 9, 10
	GWHGAOPM038577	Nuc	Chr18: 29.8	AP2×1	2, 5, 6, 7, 9, 10
	GWHGAOPM020913	Nuc	Chr10: 66.81	AP2×1	2, 5, 6, 7, 9, 10
	GWHGAOPM038579	Nuc	Chr18: 29.84	AP2×1	2, 5, 6, 7, 9, 10
	GWHGAOPM002784	Nuc	Chr02: 16.31	AP2×1	2, 5, 6, 7, 9, 10
	GWHGAOPM036604	Nuc	Chr17: 26.02	AP2×1	2, 5, 6, 7, 9, 10
	GWHGAOPM038578	Nuc	Chr18: 29.83	AP2×1	2, 5, 6, 7, 9, 10
	GWHGAOPM037294	Nuc	Chr17: 42.26	AP2×1	2, 5, 6, 9, 10
	GWHGAOPM019261	Nuc	Chr10: 40.19	AP2×1	5, 6, 7, 10
	GWHGAOPM030954	Cyt	Chr14: 35.04	AP2×1	5, 6, 7, 10
	GWHGAOPM020916	Nuc	Chr10: 66.85	AP2×1	2, 5, 6, 7, 9, 10
	GWHGAOPM020918	Nuc	Chr10: 66.87	AP2×1	2, 5, 6, 7, 9, 10
	GWHGAOPM020926	Nuc	Chr10: 67.01	AP2×1	2, 5, 6, 7, 9, 10
	GWHGAOPM020919	Nuc	Chr10: 66.89	AP2×1	2, 5, 6, 7, 9, 10
B4	GWHGAOPM029999	Nuc	Chr14: 12.57	AP2×1	2, 5, 6, 7
	GWHGAOPM053191	Nuc	Contig01243: 0.02	AP2×1	2, 5, 6, 7
	GWHGAOPM050199	Nuc	Contig001272: 0.08	AP2×1	2, 5, 6, 7
	GWHGAOPM053190	Nuc	Contig01243:	AP2×1	2, 5, 6, 7

			0.21		
	GWHGAOPM050200	Nuc	Contig001272: 0.1	AP2×1	2, 5, 6, 7
	GWHGAOPM035982	Nuc	Chr17: 12.38	AP2×1	2, 5, 6, 7
	GWHGAOPM012443	Nuc	Chr07: 4.36	AP2×1	2, 5, 6, 7
	GWHGAOPM053192	Nuc	Contig01243: 0.04	AP2×1	2, 5, 6, 7
	GWHGAOPM050198	Nuc	Contig001272: 0.07	AP2×1	2, 5, 6, 7
	GWHGAOPM046757	Nuc	Chr22: 34.68	AP2×1	2, 5, 6, 7, 8
	GWHGAOPM007344	Nuc	Chr04: 27.51	AP2×1	2, 5, 6, 7
	GWHGAOPM029486	Nuc	Chr14: 4.34	AP2×1	2, 5, 6, 7, 8
	GWHGAOPM014088	Nuc	Chr07: 38.92	AP2×1	2, 5, 6, 7
	GWHGAOPM019005	Nuc	Chr10: 36.5	AP2×1	2, 5, 6, 7, 8
	GWHGAOPM048132	Nuc	Chr23: 21.68	AP2×1	2, 5, 6, 7, 8
	GWHGAOPM015109	Nuc	Chr08: 19.01	AP2×1	2, 5, 6, 7
	GWHGAOPM010549	Nuc	Chr06: 25.14	AP2×1	2, 5, 6, 7
	GWHGAOPM048491	Nuc	Chr23: 31.84	AP2×1	2, 5, 6, 7
	GWHGAOPM038814	Nuc	Chr18: 34.1	AP2×1	2, 5, 6, 7
	GWHGAOPM037155	Nuc	Chr17: 38.97	AP2×1	2, 5, 6, 7
	GWHGAOPM037154	Nuc	Chr17: 38.97	AP2×1	2, 5, 6, 7
	GWHGAOPM002452	Nuc	Chr02: 5.66	AP2×1	2, 5, 6, 7
	GWHGAOPM002474	Nuc	Chr02: 5.96	AP2×1	2, 5, 6, 7
	GWHGAOPM039215	Nuc	Chr18: 41.19	AP2×1	5, 6, 7
	GWHGAOPM039216	Nuc	Chr18: 41.21	AP2×1	2, 5, 6, 7
	GWHGAOPM032612	Nuc	Chr15: 33.54	AP2×1	2, 5, 6, 7
	GWHGAOPM004403	Nuc	Chr03: 2.6	AP2×1	2, 5, 6, 7
	GWHGAOPM005670	Nuc	Chr03: 30.15	AP2×1	1, 2, 5, 6, 7
B5	GWHGAOPM041835	Nuc	Chr19: 45.03	AP2×1	2, 5, 6, 7
	GWHGAOPM021157	Nuc	Chr11: 2.94	AP2×1	2, 5, 6, 7
	GWHGAOPM053462	Nuc	Contig01249: 0.03	AP2×1	2, 5, 6, 7
	GWHGAOPM049909	Nuc	Contig001266: 0.01	AP2×1	2, 5, 6, 7
	GWHGAOPM000986	Nuc	Chr01: 15.48	AP2×1	2, 5, 6, 7
	GWHGAOPM026109	Nuc	Chr12: 37.61	AP2×1	2, 5, 6, 7
	GWHGAOPM000008	Cyt/Nuc	Chr01: 0.21	AP2×1	2, 5, 7, 10
	GWHGAOPM028373	Nuc	Chr13: 31.44	AP2×1	2, 5, 6, 7
	GWHGAOPM028384	Nuc	Chr13: 31.62	AP2×1	2, 5, 6, 7
	GWHGAOPM040824	Cyt/Nuc	Chr19: 20.87	AP2×1	2, 5, 6, 7
	GWHGAOPM007896	Nuc	Chr04: 37.61	AP2×1	2, 5, 6, 7
	GWHGAOPM046369	Cyt/Nuc	Chr22: 26.15	AP2×1	2, 4, 5, 6, 7
	GWHGAOPM031037	Nuc	Chr15: 1.17	AP2×1	2, 5, 6, 7
	GWHGAOPM045403	Nuc	Chr22: 4.27	AP2×1	2, 5, 6, 7

	GWHGAOPM006363	Nuc	Chr04: 5.57	AP2×1	2, 5, 6, 7
	GWHGAOPM013092	Nuc	Chr07: 20.91	AP2×1	1, 5, 6
B6	GWHGAOPM036459	Nuc	Chr17: 22.26	AP2×1	2, 5, 6, 7
	GWHGAOPM002914	Nuc	Chr02: 20.71	AP2×1	2, 5, 6, 7
	GWHGAOPM017850	Nuc	Chr10: 13.29	AP2×1	2, 5, 6, 7
	GWHGAOPM024511	Nuc	Chr12: 8.79	AP2×1	2, 5, 6, 7
	GWHGAOPM036458	Nuc	Chr17: 22.21	AP2×1	2, 5, 6, 7
	GWHGAOPM000339	Nuc	Chr01: 5.19	AP2×1	2, 5, 6, 7
	GWHGAOPM027834	Nuc	Chr13: 19.26	AP2×1	2, 5, 6, 7
	GWHGAOPM035852	Nuc	Chr17: 10.3	AP2×1	5, 6, 7
	GWHGAOPM027906	Nuc	Chr13: 20.63	AP2×1	2, 5, 6, 7
	GWHGAOPM027914	Nuc	Chr13: 20.8	AP2×1	6, 7
	GWHGAOPM046694	Nuc	Chr22: 33.58	AP2×1	2, 5, 6, 7
	GWHGAOPM007446	Nuc	Chr04: 29.55	AP2×1	2, 5, 6, 7
	GWHGAOPM040516	Nuc	Chr19: 15.57	AP2×1	2, 5, 6, 7
	GWHGAOPM035932	Nuc	Chr17: 11.55	AP2×1	2, 5, 6, 7
	GWHGAOPM018617	Cyt	Chr10: 29.61	AP2×1	2, 5, 6, 7
	GWHGAOPM004196	Nuc	Chr02: 44.58	AP2×1	2, 5, 6, 7
	GWHGAOPM004220	Nuc	Chr02: 44.87	AP2×1	2, 5, 6, 7
	GWHGAOPM004224	Cyt/Nuc	Chr02: 44.93	AP2×1	5, 6, 7
	GWHGAOPM004191	Nuc	Chr02: 44.51	AP2×1	2, 5, 6, 7
	GWHGAOPM036868	Nuc	Chr17: 33.08	AP2×1	2, 5, 6, 7
	GWHGAOPM020162	Nuc	Chr10: 52.08	AP2×1	2, 5, 6, 7
	GWHGAOPM036865	Nuc	Chr17: 33.01	AP2×1	2, 5, 6, 7
	GWHGAOPM002488	Nuc	Chr02: 6.26	AP2×1	2, 5, 6, 7
	GWHGAOPM045116	Nuc	Chr22: 0.72	AP2×1	6, 7
	GWHGAOPM006067	Nuc	Chr04: 1.05	AP2×1	6, 7
	GWHGAOPM006066	Nuc	Chr04: 1.04	AP2×1	6, 7
	GWHGAOPM052923	Nuc	Contig01238: 0.09	AP2×1	2, 5, 6, 7
	GWHGAOPM049130	Nuc	Contig001253: 0.12	AP2×1	2, 5, 6, 7
	GWHGAOPM049131	Nuc	Contig001253: 0.13	AP2×1	2, 5, 6, 7
	GWHGAOPM052924	Nuc	Contig01238: 0.09	AP2×1	2, 5, 6, 7
	GWHGAOPM047836	Nuc	Chr23: 15.7	AP2×1	2, 5, 6, 7
	GWHGAOPM005205	Nuc	Chr03: 16.76	AP2×1	2, 5, 6, 7
	GWHGAOPM004702	Cyt/Nuc	Chr03: 6.53	AP2×1	2, 5, 6, 7
	GWHGAOPM049716	Nuc	Contig001257: 1.35	AP2×1	2, 5, 6, 7
	GWHGAOPM032981	Nuc	Chr15: 39.57	AP2×1	2, 5, 6, 7
	GWHGAOPM050911	Nuc	Contig00200: 1.35	AP2×1	2, 5, 6, 7

			GWHGAOPM011024	Cyt/Nuc	Chr06: 36.43	AP2×1	5, 6
			GWHGAOPM011017	Cyt/Nuc	Chr06: 36.38	AP2×1	5, 6
			GWHGAOPM015594	Cyt/Nuc	Chr08: 27.67	AP2×1	5, 6
			GWHGAOPM011022	Cyt/Nuc	Chr06: 36.41	AP2×1	2, 5, 6
			GWHGAOPM015591	Cyt/Nuc	Chr08: 27.63	AP2×1	2, 5, 6, 7
			GWHGAOPM001547	Cyt/Nuc	Chr01: 25.52	AP2×1	2, 5, 6
			GWHGAOPM052073	Cyt/Nuc	Contig01223: 0.03	AP2×1	2, 5, 6
			GWHGAOPM021896	Cyt/Nuc	Chr11: 13.58	AP2×1	2, 5, 6
			GWHGAOPM042478	Cyt	Chr20: 5.89	AP2×1	2, 4, 5
			GWHGAOPM026279	Cyt/Nuc	Chr12: 42.92	AP2×1	2, 5, 6
RAV	-	-	GWHGAOPM010989	Nuc	Chr06: 35.9	AP2×1+B3×1	2, 5, 6, 7
			GWHGAOPM015562	Nuc	Chr08: 27.17	AP2×1+B3×1	2, 5, 6, 7
			GWHGAOPM000109	Nuc	Chr01: 1.68	AP2×1+B3×1	2, 5, 6, 7
			GWHGAOPM023375	Nuc	Chr11: 45.06	AP2×1+B3×1	2, 5, 6, 7
Soloist	-	-	GWHGAOPM045315	Nuc	Chr22: 3.1	AP2×1	2, 6
			GWHGAOPM043105	Nuc	Chr20: 21.42	AP2×1	2, 6
			GWHGAOPM006278	Nuc	Chr04: 4.25	AP2×1	2, 6

1: Nuc, Cyt, and Chl represent subcellular prediction results of nucleus, cytochrome, and chloroplast, respectively.

2: More than 80% of genes within each group contain motifs that are considered characteristic motifs of that group.

Table S2. The gene duplication within the olive AP2/ERF superfamily.

Gene pairs	Gene 1 ID	Location/Mb	Gene 2 ID	Location/Mb	Gene 3 ID	Location/Mb	Gene 4 ID	Location/Mb	Gene 5 ID	Location/Mb	Gene 6 ID	Location/Mb	Gene 7 ID	Location/Mb
Tandem duplication	GWHGAOPM000005	Chr01: 0.09	GWHGAOPM000008	Chr01: 0.21										
	GWHGAOPM000775	Chr01: 11.85	GWHGAOPM000784	Chr01: 12.02										
	GWHGAOPM000848	Chr01: 12.85	GWHGAOPM000849	Chr01: 12.85										
	GWHGAOPM001940	Chr01: 35.57	GWHGAOPM001944	Chr01: 35.64										
	GWHGAOPM003821	Chr02: 38.77	GWHGAOPM003831	Chr02: 39.12	GWHGAOPM003835	Chr02: 39.19								
	GWHGAOPM003956	Chr02: 41	GWHGAOPM003957	Chr02: 41.03										
	GWHGAOPM004191	Chr02: 44.51	GWHGAOPM004196	Chr02: 44.58										
	GWHGAOPM004220	Chr02: 44.87	GWHGAOPM004224	Chr02: 44.93										
	GWHGAOPM006066	Chr04: 1.04	GWHGAOPM006067	Chr04: 1.05										
	GWHGAOPM006966	Chr04: 16.39	GWHGAOPM006970	Chr04: 16.48	GWHGAOPM006975	Chr04: 16.56								
	GWHGAOPM007130	Chr04: 20.83	GWHGAOPM007131	Chr04: 20.88	GWHGA	Chr04: 20.91								

				OPM007132			
GWHGAOPM007569	Chr04: 31.82	GWHGAOPM007570	Chr04: 31.84				
GWHGAOPM007790	Chr04: 36.12	GWHGAOPM007797	Chr04: 36.22				
GWHGAOPM007896	Chr04: 37.61	GWHGAOPM007906	Chr04: 37.74				
GWHGAOPM008141	Chr05: 6.28	GWHGAOPM008142	Chr05: 6.28	GWHGAOPM008145	Chr05: 6.33		
GWHGAOPM008743	Chr05: 19.46	GWHGAOPM008744	Chr05: 19.48	GWHGAOPM008747	Chr05: 19.53	GWHGAOPM008748	Chr05: 19.64
GWHGAOPM009118	Chr05: 24.99	GWHGAOPM009119	Chr05: 25	GWHGAOPM009120	Chr05: 25.01		
GWHGAOPM010543	Chr06: 24.93	GWHGAOPM010549	Chr06: 25.14				
GWHGAOPM011017	Chr06: 36.38	GWHGAOPM011022	Chr06: 36.41	GWHGAOPM011024	Chr06: 36.43		

GWHGAOPM011467	Chr06: 44.19	GWHGAOPM011468	Chr06: 44.2						
GWHGAOPM012180	Chr06: 54.62	GWHGAOPM012187	Chr06: 54.76						
GWHGAOPM012353	Chr07: 2.9	GWHGAOPM012354	Chr07: 2.93						
GWHGAOPM012435	Chr07: 4.17	GWHGAOPM012443	Chr07: 4.36						
GWHGAOPM013784	Chr07: 34	GWHGAOPM013785	Chr07: 34.01						
GWHGAOPM014026	Chr07: 37.78	GWHGAOPM014030	Chr07: 37.87						
GWHGAOPM015591	Chr08: 27.63	GWHGAOPM015594	Chr08: 27.67						
GWHGAOPM018920	Chr10: 35.12	GWHGAOPM018921	Chr10: 35.13						
GWHGAOPM020378	Chr10: 55.42	GWHGAOPM020382	Chr10: 55.56						
GWHGAOPM020913	Chr10: 66.81	GWHGAOPM020916	Chr10: 66.85	GWHGAOPM020918	Chr10: 66.87	GWHGAOPM020919	Chr10: 66.89	GWHGAOPM020926	Chr10: 67.01
GWHGAOPM021433	Chr11: 6.48	GWHGAOPM021434	Chr11: 6.51	GWHGAOPM021435	Chr11: 6.53				
GWHGAOPM023993	Chr11: 56.84	GWHGAOPM023996	Chr11: 56.92	GWHGAOPM	Chr12: 0.73				

				024049									
GWHGAOPM024335	Chr12: 5.52	GWHGAOPM024336	Chr12: 5.54	GWHGAOPM024337	Chr12: 5.55								
GWHGAOPM024963	Chr12: 14.98	GWHGAOPM024967	Chr12: 15.02										
GWHGAOPM025964	Chr12: 34.64	GWHGAOPM025969	Chr12: 34.7	GWHGAOPM025971	Chr12: 34.73								
GWHGAOPM025985	Chr12: 35	GWHGAOPM025988	Chr12: 35.02										
GWHGAOPM027125	Chr13: 7.29	GWHGAOPM027126	Chr13: 7.3										
GWHGAOPM027906	Chr13: 20.63	GWHGAOPM027914	Chr13: 20.8										
GWHGAOPM028248	Chr13: 27.94	GWHGAOPM028250	Chr13: 27.98	GWHGAOPM028254	Chr13: 28.28	GWHGAOPM028255	Chr13: 28.38	GWHGAOPM028256	Chr13: 28.49	GWHGAOPM028257	Chr13: 28.55	GWHGAOPM028261	Chr13: 28.77
GWHGAOPM028373	Chr13: 31.44	GWHGAOPM028384	Chr13: 31.62										
GWHGAOPM030794	Chr14: 32.04	GWHGAOPM030797	Chr14: 32.15	GWHGAOPM0307	Chr14: 32.15								

				98	
GWHGAOPM034923	Chr16: 32.96	GWHGAOPM034924	Chr16: 32.97	GWHGAOPM034926	Chr16: 32.99
GWHGAOPM035793	Chr17: 9.39	GWHGAOPM035798	Chr17: 9.52		
GWHGAOPM035843	Chr17: 10.2	GWHGAOPM035852	Chr17: 10.3		
GWHGAOPM036458	Chr17: 22.21	GWHGAOPM036459	Chr17: 22.26		
GWHGAOPM036865	Chr17: 33.01	GWHGAOPM036868	Chr17: 33.08		
GWHGAOPM037154	Chr17: 38.97	GWHGAOPM037155	Chr17: 38.97		
GWHGAOPM038577	Chr18: 29.8	GWHGAOPM038578	Chr18: 29.83	GWHGAOPM038579	Chr18: 29.84
GWHGAOPM039188	Chr18: 40.8	GWHGAOPM039190	Chr18: 40.87		
GWHGAOPM039422	Chr18: 43.94	GWHGAOPM039423	Chr18: 43.94		
GWHGAOPM040096	Chr19: 8.96	GWHGAOPM040107	Chr19: 9.12	GWHGAOPM040108	Chr19: 9.13
GWHGAOPM041083	Chr19:	GWHGAOPM041084	Chr19:	GW	Chr19:

	26.01		26.02	HGAOPM041085	26.05
GWHGAOPM042013	Chr19: 47.94	GWHGAOPM042017	Chr19: 48		
GWHGAOPM042845	Chr20: 15.83	GWHGAOPM042851	Chr20: 16.06		
GWHGAOPM046161	Chr22: 19.46	GWHGAOPM046164	Chr22: 19.56		
GWHGAOPM046600	Chr22: 32.2	GWHGAOPM046601	Chr22: 32.24	GWHGAOPM046602	Chr22: 32.24
GWHGAOPM046757	Chr22: 34.68	GWHGAOPM046762	Chr22: 34.76		
GWHGAOPM046931	Chr23: 1.67	GWHGAOPM046941	Chr23: 1.81		
GWHGAOPM049130	Contig001253: 0.12	GWHGAOPM049131	Contig001253: 0.13		
GWHGAOPM053191	Contig01243: 0.02	GWHGAOPM053192	Contig01243: 0.04		
GWHGAOPM050198	Contig001272: 0.07	GWHGAOPM050199	Contig001272: 0.08	GWHGAOPM050200	Contig001272: 0.1
GWHGAOPM052923	Contig	GWHGAOPM052924	Contig		

		01238: 0.09		01238: 0.09
Segmental duplication	GWHGAOPM001547	Chr01: 25.52	GWHGAOPM011022	Chr06: 36.41
	GWHGAOPM001547	Chr01: 25.52	GWHGAOPM015591	Chr08: 27.63
	GWHGAOPM000339	Chr01: 5.19	GWHGAOPM017850	Chr10: 13.29
	GWHGAOPM000848	Chr01: 12.85	GWHGAOPM021433	Chr11: 6.48
	GWHGAOPM001268	Chr01: 19.65	GWHGAOPM025248	Chr12: 19.43
	GWHGAOPM001250	Chr01: 19.43	GWHGAOPM025248	Chr12: 19.43
	GWHGAOPM000634	Chr01: 9.22	GWHGAOPM024042	Chr12: 0.59
	GWHGAOPM000784	Chr01: 12.02	GWHGAOPM024190	Chr12: 2.57
	GWHGAOPM001940	Chr01: 35.57	GWHGAOPM025866	Chr12: 32.36
	GWHGAOPM001835	Chr01: 33.13	GWHGAOPM026038	Chr12: 35.81
	GWHGAOPM000848	Chr01: 12.85	GWHGAOPM024336	Chr12: 5.54
	GWHGAOPM000849	Chr01: 12.85	GWHGAOPM024337	Chr12: 5.55
	GWHGAOPM000986	Chr01: 15.48	GWHGAOPM026109	Chr12: 37.61
	GWHGAOPM000339	Chr01: 5.19	GWHGAOPM024511	Chr12: 8.79
	GWHGAOPM001547	Chr01:	GWHGAOPM026279	Chr12:

	25.52		42.92
GWHGAOPM000339	Chr01: 5.19	GWHGAOPM035852	Chr17: 10.3
GWHGAOPM000848	Chr01: 12.85	GWHGAOPM042013	Chr19: 47.94
GWHGAOPM000986	Chr01: 15.48	GWHGAOPM041835	Chr19: 45.03
GWHGAOPM002263	Chr02: 1.63	GWHGAOPM002283	Chr02: 1.99
GWHGAOPM004196	Chr02: 44.58	GWHGAOPM004220	Chr02: 44.87
GWHGAOPM004191	Chr02: 44.51	GWHGAOPM004224	Chr02: 44.93
GWHGAOPM003831	Chr02: 39.12	GWHGAOPM006803	Chr04: 13.56
GWHGAOPM004047	Chr02: 42.26	GWHGAOPM016165	Chr09: 7.32
GWHGAOPM004196	Chr02: 44.58	GWHGAOPM018617	Chr10: 29.61
GWHGAOPM002283	Chr02: 1.99	GWHGAOPM036979	Chr17: 35.77
GWHGAOPM004047	Chr02: 42.26	GWHGAOPM035793	Chr17: 9.39
GWHGAOPM003831	Chr02: 39.12	GWHGAOPM035573	Chr17: 5.91
GWHGAOPM002452	Chr02: 5.66	GWHGAOPM037154	Chr17: 38.97
GWHGAOPM002474	Chr02: 5.96	GWHGAOPM037154	Chr17: 38.97
GWHGAOPM002914	Chr02: 20.71	GWHGAOPM036458	Chr17: 22.21

GWHGAOPM004196	Chr02: 44.58	GWHGAOPM035932	Chr17: 11.55
GWHGAOPM002488	Chr02: 6.26	GWHGAOPM036865	Chr17: 33.01
GWHGAOPM002539	Chr02: 7.46	GWHGAOPM038678	Chr18: 31.62
GWHGAOPM002452	Chr02: 5.66	GWHGAOPM038814	Chr18: 34.1
GWHGAOPM002474	Chr02: 5.96	GWHGAOPM038814	Chr18: 34.1
GWHGAOPM004776	Chr03: 7.8	GWHGAOPM005465	Chr03: 26.02
GWHGAOPM004403	Chr03: 2.6	GWHGAOPM005670	Chr03: 30.15
GWHGAOPM004702	Chr03: 6.53	GWHGAOPM005205	Chr03: 16.76
GWHGAOPM004776	Chr03: 7.8	GWHGAOPM008766	Chr05: 19.9
GWHGAOPM004882	Chr03: 9.81	GWHGAOPM033187	Chr15: 42.75
GWHGAOPM005465	Chr03: 26.02	GWHGAOPM031732	Chr15: 13.37
GWHGAOPM004776	Chr03: 7.8	GWHGAOPM031732	Chr15: 13.37
GWHGAOPM004776	Chr03: 7.8	GWHGAOPM033049	Chr15: 40.67
GWHGAOPM004403	Chr03: 2.6	GWHGAOPM032612	Chr15: 33.54
GWHGAOPM005670	Chr03: 30.15	GWHGAOPM032612	Chr15: 33.54
GWHGAOPM004702	Chr03:	GWHGAOPM032981	Chr15:

	6.53		39.57
GWHGAOPM004776	Chr03: 7.8	GWHGAOPM034407	Chr16: 24.87
GWHGAOPM005465	Chr03: 26.02	GWHGAOPM034407	Chr16: 24.87
GWHGAOPM004403	Chr03: 2.6	GWHGAOPM039216	Chr18: 41.21
GWHGAOPM007790	Chr04: 36.12	GWHGAOPM007797	Chr04: 36.22
GWHGAOPM007797	Chr04: 36.22	GWHGAOPM011827	Chr06: 49.85
GWHGAOPM007851	Chr04: 36.99	GWHGAOPM011468	Chr06: 44.2
GWHGAOPM006966	Chr04: 16.39	GWHGAOPM012435	Chr07: 4.17
GWHGAOPM007131	Chr04: 20.88	GWHGAOPM012354	Chr07: 2.93
GWHGAOPM007344	Chr04: 27.51	GWHGAOPM014088	Chr07: 38.92
GWHGAOPM007851	Chr04: 36.99	GWHGAOPM014413	Chr08: 2.02
GWHGAOPM007753	Chr04: 35.56	GWHGAOPM014774	Chr08: 9.72
GWHGAOPM007906	Chr04: 37.74	GWHGAOPM021026	Chr11: 1.14
GWHGAOPM006858	Chr04: 14.36	GWHGAOPM022681	Chr11: 26.42
GWHGAOPM007753	Chr04: 35.56	GWHGAOPM028110	Chr13: 24.08
GWHGAOPM007569	Chr04: 31.82	GWHGAOPM028254	Chr13: 28.28

GWHGAOPM007896	Chr04: 37.61	GWHGAOPM028384	Chr13: 31.62
GWHGAOPM007277	Chr04: 25.95	GWHGAOPM030456	Chr14: 23.58
GWHGAOPM006966	Chr04: 16.39	GWHGAOPM030723	Chr14: 30.33
GWHGAOPM007130	Chr04: 20.83	GWHGAOPM030797	Chr14: 32.15
GWHGAOPM007130	Chr04: 20.83	GWHGAOPM034924	Chr16: 32.97
GWHGAOPM006803	Chr04: 13.56	GWHGAOPM035573	Chr17: 5.91
GWHGAOPM007851	Chr04: 36.99	GWHGAOPM041085	Chr19: 26.05
GWHGAOPM007896	Chr04: 37.61	GWHGAOPM040824	Chr19: 20.87
GWHGAOPM006104	Chr04: 1.59	GWHGAOPM042851	Chr20: 16.06
GWHGAOPM006278	Chr04: 4.25	GWHGAOPM043105	Chr20: 21.42
GWHGAOPM007277	Chr04: 25.95	GWHGAOPM046315	Chr22: 24.88
GWHGAOPM006104	Chr04: 1.59	GWHGAOPM045147	Chr22: 1.16
GWHGAOPM007797	Chr04: 36.22	GWHGAOPM046433	Chr22: 27.66
GWHGAOPM007753	Chr04: 35.56	GWHGAOPM046464	Chr22: 28.4
GWHGAOPM006858	Chr04: 14.36	GWHGAOPM045804	Chr22: 10.41
GWHGAOPM007569	Chr04:	GWHGAOPM046601	Chr22:

	31.82		32.24
GWHGAOPM007570	Chr04: 31.84	GWHGAOPM046600	Chr22: 32.2
GWHGAOPM007130	Chr04: 20.83	GWHGAOPM046164	Chr22: 19.56
GWHGAOPM007344	Chr04: 27.51	GWHGAOPM046757	Chr22: 34.68
GWHGAOPM007896	Chr04: 37.61	GWHGAOPM046369	Chr22: 26.15
GWHGAOPM006363	Chr04: 5.57	GWHGAOPM045403	Chr22: 4.27
GWHGAOPM007446	Chr04: 29.55	GWHGAOPM046694	Chr22: 33.58
GWHGAOPM006066	Chr04: 1.04	GWHGAOPM045116	Chr22: 0.72
GWHGAOPM006278	Chr04: 4.25	GWHGAOPM045315	Chr22: 3.1
GWHGAOPM008233	Chr05: 8.73	GWHGAOPM010521	Chr06: 24.23
GWHGAOPM008141	Chr05: 6.28	GWHGAOPM012180	Chr06: 54.62
GWHGAOPM008141	Chr05: 6.28	GWHGAOPM018819	Chr10: 33.21
GWHGAOPM008766	Chr05: 19.9	GWHGAOPM033049	Chr15: 40.67
GWHGAOPM008766	Chr05: 19.9	GWHGAOPM034407	Chr16: 24.87
GWHGAOPM009118	Chr05: 24.99	GWHGAOPM034924	Chr16: 32.97
GWHGAOPM011467	Chr06: 44.19	GWHGAOPM014413	Chr08: 2.02

GWHGAOPM011827	Chr06: 49.85	GWHGAOPM014861	Chr08: 11.61
GWHGAOPM010688	Chr06: 28.89	GWHGAOPM015249	Chr08: 21.4
GWHGAOPM010549	Chr06: 25.14	GWHGAOPM015109	Chr08: 19.01
GWHGAOPM011024	Chr06: 36.43	GWHGAOPM015594	Chr08: 27.67
GWHGAOPM011022	Chr06: 36.41	GWHGAOPM015591	Chr08: 27.63
GWHGAOPM010989	Chr06: 35.9	GWHGAOPM015562	Chr08: 27.17
GWHGAOPM010688	Chr06: 28.89	GWHGAOPM022265	Chr11: 19.53
GWHGAOPM011022	Chr06: 36.41	GWHGAOPM021896	Chr11: 13.58
GWHGAOPM010543	Chr06: 24.93	GWHGAOPM026423	Chr12: 46.54
GWHGAOPM010435	Chr06: 21.15	GWHGAOPM027125	Chr13: 7.29
GWHGAOPM011827	Chr06: 49.85	GWHGAOPM037820	Chr18: 9.94
GWHGAOPM011468	Chr06: 44.2	GWHGAOPM041085	Chr19: 26.05
GWHGAOPM010435	Chr06: 21.15	GWHGAOPM040107	Chr19: 9.12
GWHGAOPM010435	Chr06: 21.15	GWHGAOPM046941	Chr23: 1.81
GWHGAOPM013152	Chr07: 22.34	GWHGAOPM020124	Chr10: 51.65
GWHGAOPM014206	Chr07:	GWHGAOPM018920	Chr10:

	40.62		35.12
GWHGAOPM014088	Chr07: 38.92	GWHGAOPM019005	Chr10: 36.5
GWHGAOPM013232	Chr07: 24.15	GWHGAOPM030136	Chr14: 15.1
GWHGAOPM014206	Chr07: 40.62	GWHGAOPM029369	Chr14: 2.47
GWHGAOPM012435	Chr07: 4.17	GWHGAOPM030723	Chr14: 30.33
GWHGAOPM014026	Chr07: 37.78	GWHGAOPM029550	Chr14: 5.24
GWHGAOPM012353	Chr07: 2.9	GWHGAOPM030797	Chr14: 32.15
GWHGAOPM012354	Chr07: 2.93	GWHGAOPM030794	Chr14: 32.04
GWHGAOPM014088	Chr07: 38.92	GWHGAOPM029486	Chr14: 4.34
GWHGAOPM013152	Chr07: 22.34	GWHGAOPM036840	Chr17: 32.4
GWHGAOPM013152	Chr07: 22.34	GWHGAOPM039422	Chr18: 43.94
GWHGAOPM012353	Chr07: 2.9	GWHGAOPM046164	Chr22: 19.56
GWHGAOPM012354	Chr07: 2.93	GWHGAOPM046161	Chr22: 19.46
GWHGAOPM014088	Chr07: 38.92	GWHGAOPM048132	Chr23: 21.68
GWHGAOPM015249	Chr08: 21.4	GWHGAOPM022265	Chr11: 19.53
GWHGAOPM015591	Chr08: 27.63	GWHGAOPM021896	Chr11: 13.58

GWHGAOPM015591	Chr08: 27.63	GWHGAOPM026279	Chr12: 42.92
GWHGAOPM014774	Chr08: 9.72	GWHGAOPM028110	Chr13: 24.08
GWHGAOPM014413	Chr08: 2.02	GWHGAOPM041085	Chr19: 26.05
GWHGAOPM014774	Chr08: 9.72	GWHGAOPM046464	Chr22: 28.4
GWHGAOPM016165	Chr09: 7.32	GWHGAOPM028960	Chr13: 46.51
GWHGAOPM017031	Chr09: 28.96	GWHGAOPM042652	Chr20: 9.48
GWHGAOPM019285	Chr10: 40.64	GWHGAOPM023303	Chr11: 42.95
GWHGAOPM017850	Chr10: 13.29	GWHGAOPM024511	Chr12: 8.79
GWHGAOPM020470	Chr10: 56.89	GWHGAOPM030456	Chr14: 23.58
GWHGAOPM020382	Chr10: 55.56	GWHGAOPM030136	Chr14: 15.1
GWHGAOPM018921	Chr10: 35.13	GWHGAOPM029369	Chr14: 2.47
GWHGAOPM020745	Chr10: 61.39	GWHGAOPM029550	Chr14: 5.24
GWHGAOPM019005	Chr10: 36.5	GWHGAOPM029486	Chr14: 4.34
GWHGAOPM020124	Chr10: 51.65	GWHGAOPM036840	Chr17: 32.4
GWHGAOPM020378	Chr10: 55.42	GWHGAOPM036979	Chr17: 35.77
GWHGAOPM020745	Chr10:	GWHGAOPM037199	Chr17:

	61.39		40.12
GWHGAOPM018617	Chr10: 29.61	GWHGAOPM035932	Chr17: 11.55
GWHGAOPM020162	Chr10: 52.08	GWHGAOPM036865	Chr17: 33.01
GWHGAOPM020470	Chr10: 56.89	GWHGAOPM039086	Chr18: 38.97
GWHGAOPM020378	Chr10: 55.42	GWHGAOPM039190	Chr18: 40.87
GWHGAOPM020382	Chr10: 55.56	GWHGAOPM039188	Chr18: 40.8
GWHGAOPM020745	Chr10: 61.39	GWHGAOPM038759	Chr18: 33.26
GWHGAOPM017938	Chr10: 15.85	GWHGAOPM037962	Chr18: 12.77
GWHGAOPM020926	Chr10: 67.01	GWHGAOPM038577	Chr18: 29.79
GWHGAOPM020913	Chr10: 66.81	GWHGAOPM038577	Chr18: 29.79
GWHGAOPM020916	Chr10: 66.85	GWHGAOPM038578	Chr18: 29.83
GWHGAOPM019285	Chr10: 40.64	GWHGAOPM047585	Chr23: 11.64
GWHGAOPM019285	Chr10: 40.64	GWHGAOPM047562	Chr23: 11.4
GWHGAOPM018920	Chr10: 35.12	GWHGAOPM048197	Chr23: 23.18
GWHGAOPM019488	Chr10: 43.19	GWHGAOPM047342	Chr23: 7.55
GWHGAOPM019005	Chr10: 36.5	GWHGAOPM048132	Chr23: 21.68

GWHGAOPM022681	Chr11: 26.42	GWHGAOPM024963	Chr12: 14.98
GWHGAOPM021157	Chr11: 2.94	GWHGAOPM026109	Chr12: 37.61
GWHGAOPM021157	Chr11: 2.94	GWHGAOPM031037	Chr15: 1.17
GWHGAOPM022794	Chr11: 28.92	GWHGAOPM034923	Chr16: 32.96
GWHGAOPM021026	Chr11: 1.14	GWHGAOPM041692	Chr19: 42.71
GWHGAOPM021434	Chr11: 6.51	GWHGAOPM042013	Chr19: 47.94
GWHGAOPM021435	Chr11: 6.53	GWHGAOPM042017	Chr19: 48
GWHGAOPM021157	Chr11: 2.94	GWHGAOPM041835	Chr19: 45.03
GWHGAOPM022567	Chr11: 24.32	GWHGAOPM043453	Chr20: 28.6
GWHGAOPM021896	Chr11: 13.58	GWHGAOPM042478	Chr20: 5.89
GWHGAOPM023303	Chr11: 42.95	GWHGAOPM047585	Chr23: 11.64
GWHGAOPM025964	Chr12: 34.64	GWHGAOPM025985	Chr12: 35
GWHGAOPM026109	Chr12: 37.61	GWHGAOPM041835	Chr19: 45.03
GWHGAOPM027304	Chr13: 9.99	GWHGAOPM039967	Chr19: 7.1
GWHGAOPM028491	Chr13: 34.06	GWHGAOPM041320	Chr19: 32.27
GWHGAOPM027125	Chr13:	GWHGAOPM040107	Chr19:

	7.29		9.12
GWHGAOPM026822	Chr13: 3.38	GWHGAOPM039870	Chr19: 4.88
GWHGAOPM028262	Chr13: 28.84	GWHGAOPM041144	Chr19: 27.09
GWHGAOPM027700	Chr13: 16.95	GWHGAOPM040299	Chr19: 12.06
GWHGAOPM027906	Chr13: 20.63	GWHGAOPM040516	Chr19: 15.57
GWHGAOPM027914	Chr13: 20.8	GWHGAOPM040516	Chr19: 15.57
GWHGAOPM026822	Chr13: 3.38	GWHGAOPM044695	Chr21: 21.81
GWHGAOPM028384	Chr13: 31.62	GWHGAOPM046369	Chr22: 26.15
GWHGAOPM028491	Chr13: 34.06	GWHGAOPM047999	Chr23: 19.05
GWHGAOPM027126	Chr13: 7.3	GWHGAOPM046941	Chr23: 1.81
GWHGAOPM029999	Chr14: 12.57	GWHGAOPM037155	Chr17: 38.97
GWHGAOPM030136	Chr14: 15.1	GWHGAOPM039188	Chr18: 40.8
GWHGAOPM029550	Chr14: 5.24	GWHGAOPM038759	Chr18: 33.26
GWHGAOPM030797	Chr14: 32.15	GWHGAOPM046164	Chr22: 19.56
GWHGAOPM030794	Chr14: 32.04	GWHGAOPM046161	Chr22: 19.46
GWHGAOPM029486	Chr14: 4.34	GWHGAOPM048132	Chr23: 21.68

GWHGAOPM031732	Chr15: 13.37	GWHGAOPM034407	Chr16: 24.87
GWHGAOPM033049	Chr15: 40.67	GWHGAOPM034407	Chr16: 24.87
GWHGAOPM032612	Chr15: 33.54	GWHGAOPM039215	Chr18: 41.19
GWHGAOPM034924	Chr16: 32.97	GWHGAOPM046164	Chr22: 19.56
GWHGAOPM034923	Chr16: 32.96	GWHGAOPM046161	Chr22: 19.46
GWHGAOPM036840	Chr17: 32.4	GWHGAOPM039423	Chr18: 43.94
GWHGAOPM036979	Chr17: 35.77	GWHGAOPM039190	Chr18: 40.87
GWHGAOPM037199	Chr17: 40.12	GWHGAOPM038759	Chr18: 33.26
GWHGAOPM037154	Chr17: 38.97	GWHGAOPM038814	Chr18: 34.1
GWHGAOPM035982	Chr17: 12.38	GWHGAOPM048491	Chr23: 31.84
GWHGAOPM039870	Chr19: 4.88	GWHGAOPM044695	Chr21: 21.81
GWHGAOPM040107	Chr19: 9.12	GWHGAOPM046941	Chr23: 1.81
GWHGAOPM040096	Chr19: 8.96	GWHGAOPM046931	Chr23: 1.67
GWHGAOPM042851	Chr20: 16.06	GWHGAOPM045147	Chr22: 1.16
GWHGAOPM043105	Chr20: 21.42	GWHGAOPM045315	Chr22: 3.1
GWHGAOPM047562	Chr23:	GWHGAOPM047585	Chr23:

GWHGAOPM004047	11.4<br>Chr02:<br>42.26	GWHGAOPM016165	11.64<br>Chr09:<br>7.32

Table S3. The orthologs in *A. thaliana* and potential protein-protein interactions (PPIs) of candidate AP2/ERF proteins in olive.

Candidate proteins	Orthologs in *A. thaliana*	Sequence similarity/%	E-value	Predicted PPIs
GWHGAOPM041144	AT5G47220	46.3	2.00E-42	AT3G23240/AT4G36920/ACO4/EIL1/EIL2/EIL3/EIL4/EIL5/PDF1.2A
GWHGAOPM012354	AT3G23240	62.63	8.00E-63	AT4G36920/AT5G47220/ACO4/EIL1/EIL2/EIL3/EIL4/EIL5/PDF1.2A
GWHGAOPM030794		64.36	2.00E-62	
GWHGAOPM007131		51.79	6.00E-47	
GWHGAOPM046161		60.64	2.00E-54	
GWHGAOPM002263	AT4G36920	66.07	7.00E-102	AT3G23240/AT5G47220/ACO4/EIL1/PDF1.2A/PLT3
GWHGAOPM020378		65.92	4.00E-98	
GWHGAOPM045147	AT2G28550	45.96	5.00E-78	ACO4
GWHGAOPM006104		49.31	4.00E-46	
GWHGAOPM021434	AT5G47230	46.38	5.00E-47	PDF1.2A
GWHGAOPM038678	AT5G10510	56.94	1.00E-121	PLT3
GWHGAOPM002539		47.41	8.00E-105	
GWHGAOPM003646	AT5G57390	55.37	9.00E-124	PLT3/F15M7.12
GWHGAOPM010989	AT1G13260	64.07	6.00E-120	F25G13.130
GWHGAOPM023375	AT1G51120	57.92	9.00E-78	F25G13.130
GWHGAOPM042017	AT4G17500	55.56	8.00E-53	/
GWHGAOPM024335		45.28	9.00E-44	
GWHGAOPM002263	AT4G36920	66.07	7.00E-102	/
GWHGAOPM020378		65.92	4.00E-98	
GWHGAOPM016165	AT5G11590	55.79	1.00E-44	/
GWHGAOPM023996	AT1G19210	47.76	8.00E-43	/
GWHGAOPM023993		48.02	6.00E-42	
GWHGAOPM006966	AT4G13620	50	1.00E-65	/
